# A Microenvironment‐Responsive, Controlled Release Hydrogel Delivering Embelin to Promote Bone Repair of Periodontitis via Anti‐Infection and Osteo‐Immune Modulation

**DOI:** 10.1002/advs.202403786

**Published:** 2024-07-08

**Authors:** Guanming Cai, Lin Ren, Jiali Yu, Siqi Jiang, Gen Liu, Shujie Wu, Bin Cheng, Weichang Li, Juan Xia

**Affiliations:** ^1^ Hospital of Stomatology Guanghua School of Stomatology Sun Yat‐sen University Guangzhou 510055 P. R. China; ^2^ Guangdong Provincial Key Laboratory of Stomatology Guangzhou 510055 China

**Keywords:** bone regeneration, drug delivery, embelin, hydrogel, periodontitis

## Abstract

Periodontitis, a prevalent chronic inflammatory disease, poses significant challenges for effective treatment due to its complex etiology involving specific bacteria and the inflammatory immune microenvironment. Here, this study presents a novel approach for the targeted treatment of periodontitis utilizing the immunomodulatory and antibacterial properties of Embelin, a plant‐derived compound, within an injectable hydrogel system. The developed Carboxymethyl Chitosan‐Oxidized Dextran (CMCS‐OD) hydrogel formed via dynamic chemical bonds exhibited self‐healing capabilities and pH‐responsive behavior, thereby facilitating the controlled release of Embelin and enhancing its efficacy in a dynamic oral periodontitis microenvironment. This study demonstrates that this hydrogel system effectively prevents bacterial invasion and mitigates excessive immune response activation. Moreover, it precisely modulates macrophage M1/M2 phenotypes and suppresses inflammatory cytokine expression, thereby fostering a conducive environment for bone regeneration and addressing periodontitis‐induced bone loss. These findings highlight the potential of the approach as a promising strategy for the clinical management of periodontitis‐induced bone destruction.

## Graphical abstract

This study presents a novel microenvironment‐responsive hydrogel system combining immunomodulatory and antibacterial properties of Embelin for targeted periodontitis treatment. The self‐healing, pH‐responsive Carboxymethyl Chitosan‐Oxidized Dextran hydrogel ensures controlled Embelin release, effectively preventing bacterial invasion, modulating immune response, and promoting bone regeneration in periodontitis‐affected areas.

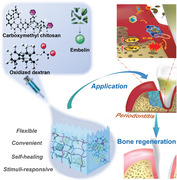

## 1 Introduction

Periodontitis is the second most prevalent oral disease, affecting 19% of the global adult population and imparting a large socioeconomic burden. It is characterized by the progressive destruction of periodontal tissues, which leads to the loss of teeth and masticatory dysfunction.^[^ ^1^ ^]^ Moreover, periodontitis is closely related to the progression of systemic diseases such as atherosclerotic disease,^[^ ^2^ ^]^ thus, seriously affecting the overall health of patients. Traditionally, treatments aim to remove dental plaque through scaling or root planning. Clinically, the treatment of periodontitis remains challenging owing to frequent revisits, recurrence, and poor prognosis.^[^ ^3^ ^]^ Adjunctive therapies, such as antibiotics, help control infection but result in drug resistance, microbial imbalance, and risk of systemic toxicity.^[^ ^4^ ^]^ Although guided tissue regeneration may be a viable substitute for the reconstruction of periodontal tissues, it has unpredictable bone regeneration results owing to its contact inhibition of epithelial growth and lack of inherent bioactivity.^[^ ^5^ ^]^ Recent advances in hydrogels have attracted substantial attention in the field of periodontitis owing to their biocompatibility, biodegradability, and accessibility for bioactive factor delivery. Functioning as a drug delivery platform, hydrogels have the potential to treat inflammatory diseases, while simultaneously serving as scaffolds to promote tissue regeneration.^[^ ^6^ ^]^ However, their unstable bioactivity in the oral environment and complex production processes hinder their clinical application.^[^ ^7^ ^]^

To ensure sufficient bioactivity of hydrogel delivery systems, it is crucial to include appropriate bioactive agents that effectively target infections and inflammation. Growing evidence indicates that periodontitis is heavily implicated in periodontal microbial dysbiosis, wherein *Porphyromonas gingivalis* (*P. gingivalis*) plays a key role in disturbing the equilibrium of periodontal immunity.^[^ ^1^, ^8^ ^]^ Toll‐like receptor pathways are subsequently activated,^[^ ^9^ ^]^ which further leads to the activation of pro‐inflammatory macrophages (M1 phenotype) and subsequent Th17 cells.^[^ ^10^, ^11^ ^]^ Repeated infections and persistent immune abnormalities trigger a storm of inflammatory cytokines, which eventually contribute to the destruction of the periodontal tissue.^[^ ^12^, ^13^ ^]^

Embelin (Emb), a small‐molecule bioactive factor extracted from the *Embelia ribes* plant,^[^ ^14^, ^15^, ^16^, ^17^, ^18^, ^19^ ^]^ is endowed with multiple bioactivities including immunomodulation,^[^ ^16^ ^]^ metabolic regulation, and antioxidant capacity,^[^ ^17^, ^18^ ^]^ which have been reported to exert positive effects on acute liver injury and antiatherogenic.^[^ ^17^, ^18^ ^]^ Moreover, Emb alleviates pathological renal damage by promoting M1‐M2 phenotype transformation of bone marrow‐derived macrophages (BMDM).^[^ ^16^ ^]^ Additionally, Emb exhibits outstanding antimicrobial capabilities against multidrug‐resistant bacteria in vitro.^[^ ^14^ ^]^ Inspiringly, the differentiation of RAW264.7 into osteoclasts can be impeded by Emb,^[^ ^19^ ^]^ indicating its potential for application in bone resorption‐related diseases. Compared with peptides or synthetic small molecules, Emb is a cost‐effective, structurally stable, and easy‐to‐prepare agent.^[^ ^20^ ^]^ Due to these features, Emb has been proposed as an outstanding candidate for reinforcing the bioactivity of hydrogel delivery systems to combat infections and immune dysregulation in periodontitis. However, Emb has low solubility when administered locally to treat periodontitis.

To maximize the effectiveness of Emb in treating periodontitis, we designed an injectable Carboxymethyl Chitosan (CMCS‐OD) based on available CMCS and Oxidized Dextran (ODex). This hydrogel exhibits broad antimicrobial properties, biocompatibility, and biodegradability.^[^ ^21^, ^22^, ^23^ ^]^ Particularly, the pH‐sensitive ability of the hydrogel enabled Emb to be released from specifically targeted acidic microenvironments of periodontitis lesions.^[^ ^24^ ^]^ In addition, the hydrogel possesses self‐healing properties that allow it to rebind following the fragmentation of deep periodontal pockets.^[^ ^25^ ^]^ This characteristic guarantees the convenience of application in periodontal tissue and the stability of Emb release in situ. Furthermore, under mild conditions, the aldehyde groups of ODex easily react with the amino groups of CMCS to form Schiff base linkages, thereby simplifying the preparation of the hydrogel delivery system.

**Figure 1:**
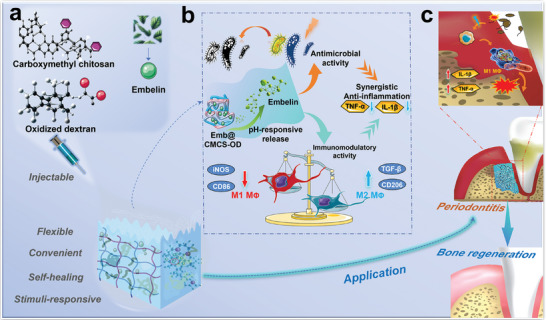
The schematic illustration of the preparation and bone regeneration promotion mechanism of Emb@CMCS‐OD. a) Synthetic process and structure representation of Emb@CMCS‐OD. b,c) The mechanism of Emb@CMCS‐OD for promoting periodontal bone regeneration. (During the onset of periodontitis, pathogenic infections disturb the periodontal immune system, which further leads to the activation of pro‐inflammatory macrophages (M1 phenotype). Repeated infections and persistent immune abnormalities trigger a cytokine storm, eventually contributing to destruction of periodontal tissue. Emb@CMCS‐OD was designed to address microbial infection and rectify immune imbalances, especially hindering the infection by *P. gingivalis*, stimulating the shift of macrophage from the M1 to M2 phenotype and suppressing the expression of TNF‐*α* and IL‐1*β*, thereby promoting the regeneration of periodontal bone).

In this study, a multifunctional drug delivery system based on Emb and CMCS‐OD was developed. Selecting an appropriate concentration of the reaction system is crucial for the performance of the hydrogels when preparing the CMCS‐OD multifunctional hydrogels. This selection is influenced not only by the results of previous studies but also by various parameters such as molecular weight, viscosity, and degree of substitution, which collectively determine the gelation characteristics,^[^ ^26^ ^]^ biocompatibility,^[^ ^27^ ^]^ and bioactivity of the hydrogels.^[^ ^26^ ^]^ Therefore, we decided to use a working concentration of 4% CMCS and 2% OD to prepare CMCS‐OD based on the concentration range reported in previous studies and experimental observations. This concentration choice aims to balance the chemical and physical properties of the hydrogel as well as its efficacy and safety in biomedical applications. This system was designed to address microbial infection and rectify immune imbalances, especially by hindering the infection of *P. gingivalis* and stimulating the shift of macrophages from a pro‐inflammatory to an anti‐inflammatory phenotype, thereby promoting the regeneration of periodontal bone (**Figure** **1**). This approach effectively alleviated periodontal inflammation and promoted bone regeneration, and provided a convenient, efficient, and promising treatment strategy for periodontitis.

## 2 Results and Discussion

### 2.1 Formation and Characterization of Emb@CMCS‐OD

**Figure 2:**
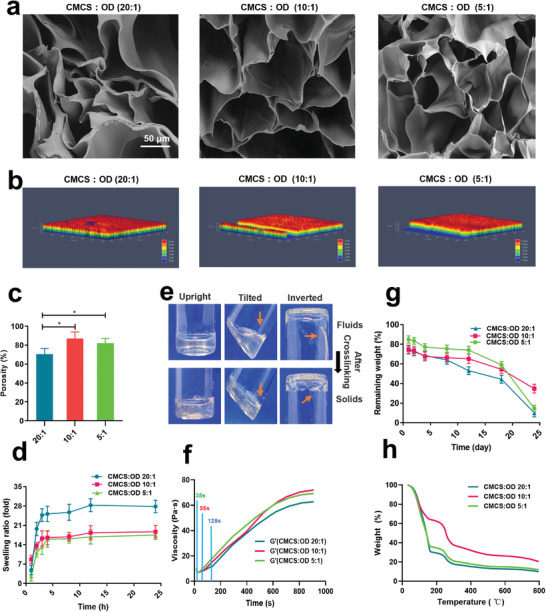
Characterization and properties of CMCS‐OD. a) SEM images of CMCS‐OD. b) Surface roughness analysis of CMCS‐OD. c) The porosity of CMCS‐OD. d) Swelling ratios of CMCS‐OD at different time points in PBS. e) The transformation of the CMCS and OD mixing solution from liquid to solid in bottles. f) Viscosity changes of CMCS‐OD pre‐hydrogel solutions over time. g) Degradability of CMCS‐OD was evaluated in phosphate‐buffered saline (PBS). h) TGA curves of CMCS‐OD. Data were expressed as mean ± SD. *n* = 3.

A multifunctional hydrogel based on CMCS and ODex was successfully prepared by dynamic chemical crosslinking, and the volume ratio of CMCS (4%) to ODex (2%) with the best performance was explored. Scanning electron microscopy (SEM) images revealed that all hydrogels exhibited a typical porous structure (**Figure** **2**a). When CMCS (40 mg mL^−1^) and ODex (20 mg mL^−1^) were mixed in ratios of 20:1 and 5:1, respectively, the hydrogel surface and internal pore structure were less regular. Meanwhile, the hydrogel with the 20:1 ratio showed a significantly decreased pore size, which may be related to insufficient support from the cross‐linked network, thereby causing pore collapse.^[^ ^28^ ^]^ In contrast, at a 10:1 ratio, the 3D structure and arrangement of the hydrogel became more uniform with reduced pore collapse (Figure 2a; Figure S1, Supporting Information). Confocal microscopy images showed that all the groups exhibited a rough surface structure (Figure 2b), which has been proven to be conducive to the adhesion and colonization of cells, particularly the orientation of osteoblasts and the M2 polarization of macrophages.^[^ ^29^ ^]^ The porosity of the 20:1 hydrogel was slightly lower, whereas that of the other two groups remained stable above 75% (Figure 2c). As shown in Figure 2d, the swelling ratios of the hydrogel at 20:1, 10:1, and 5:1 ratio were 27.84 ± 0.76, 18.71 ± 0.26, and 17.53 ± 0.66, respectively. Porosity and swelling ratio can influence the Emb loading and release rates. More specifically, a higher porosity means that more bioactive agents can be loaded per unit volume, whereas a larger swelling ratio indicates stronger hydrophilicity of the cross‐linked network and a faster release rate.^[^ ^23^ ^]^ Based on these findings, the 20:1 hydrogel had the weakest sustained‐release effect, whereas the 5:1 and 10:1 hydrogels exhibited advantages in Emb release.

As indicated in Figure 2e, the pre‐hydrogel solution in the tilted and inverted bottles had high fluidity but then exhibited a stable gel state after rapid solidification. Gelation curves,^[^ ^30^ ^]^ measured using a rheometer (Figure 2f), demonstrated that the 5:1 hydrogel experienced a rapid increase in fluid viscosity at ≈35 s. This is in contrast to the 20:1 hydrogel, which took over 2 min to gel. Importantly, only the 10:1 hydrogel achieved gelling within 55 ± 3 s. As the concentration of ODex increased, the gelation time accelerated in favor of the optimal gelation time.^[^ ^28^ ^]^ Considering the clinical administration in periodontal pockets, a duration of ≈1 min was deemed appropriate for clinical operations. Degradation experiments demonstrated that the 10:1 ratio hydrogel exhibited greater stability, degrading ≈60% of the PBS within 25 days, whereas the other two groups showed 90% degradation within the same time (Figure 2g). In addition, thermogravimetric analysis confirmed that the hydrogel with a 10:1 ratio exhibited superior thermal stability compared to the other two groups (Figure 2h).

The degradation rate of hydrogels is closely related to the crosslinking density and composition of the chemical groups. The crosslinking density of the hydrogels is a critical factor in determining their degradation rates. For instance, by altering the type and concentration of crosslinking agents, the crosslinking density of hydrogels can be adjusted, thereby affecting their mechanical properties and degradation behaviors.^[^ ^31^ ^]^ A densely crosslinked hydrogel network may lead to a slower degradation rate, whereas a lower crosslinking density may facilitate faster degradation. Moreover, specific chemical modifications can directly affect the hydrogel degradation rate.^[^ ^32^ ^]^ For example, by introducing slower‐hydrolyzing caprolactone groups or faster‐hydrolyzing lactide groups between the biopolymer backbone and the conjugated reactive groups, degradation can be controlled to match the role of the nano‐scaffolds in tissue regeneration.^[^ ^32^ ^]^ In our study, we found that different ratios of the matrix to crosslinking agent could affect the degradation rate, which was related to the aforementioned differences in crosslinking density. Further experimental exploration of other properties remains a direction for future research.

Based on the experimental results regarding the gelation rate, porosity, swelling properties, and degradation performance, we concluded that the hydrogel with a ratio of 10:1 exhibited the most comprehensive performance, including appropriate gelation time, stable degradation, thermal stability, and excellent bioactive factor sustained‐release potential. Therefore, a volumetric mixing ratio of 10:1 (CMCS:ODex) was chosen as the preparation condition for subsequent investigations of the other properties of CMCS‐OD in our future studies.

**Figure 3:**
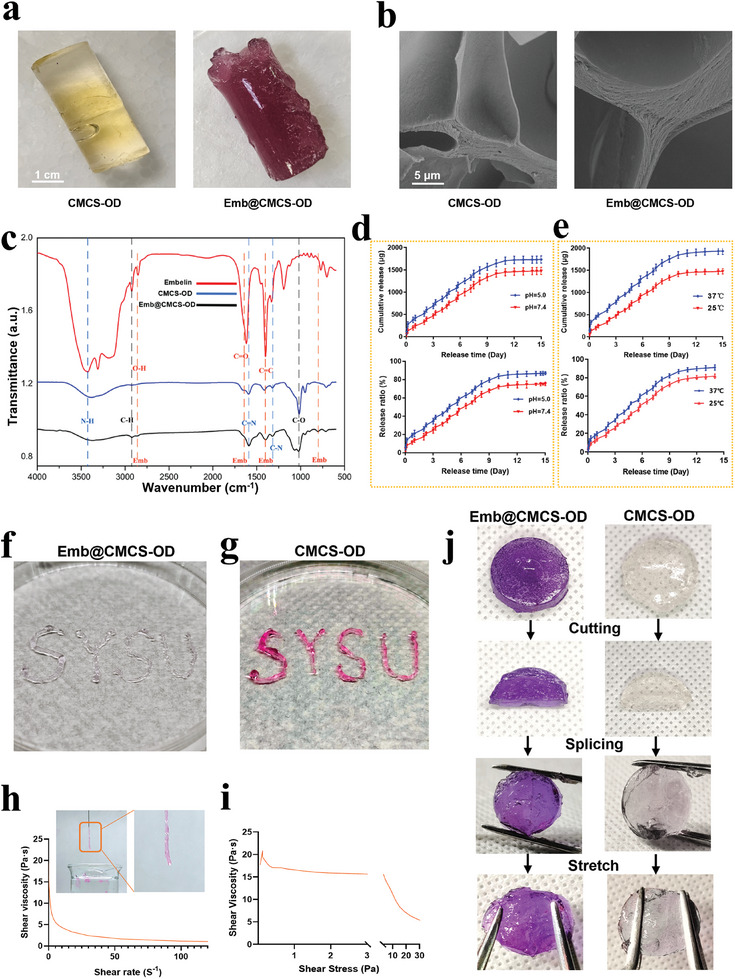
Characterization and properties of Emb@CMCS‐OD. a) Representative images of CMCS‐OD (colorless) and Emb@CMCS‐OD (mauve). b) SEM images of the CMCS‐OD surface with or without Emb. c) FT‐IR spectra of Emb, CMCS‐OD and Emb@CMCS‐OD. d,e) The release kinetics (cumulative release mass or ratio) of Emb released from Emb@CMCS‐OD at different pH (5.0 and 7.4) and temperatures (25 and 37 °C). f,g) Photographs of the letters of “SYSU”, which were written by extruding the mixing solution of Emb@CMCS‐OD/CMCS‐OD through a needle. Representative shear rate h) and shear stress i) ramps of the CMCS‐OD solution. The shear viscosity was monitored for shear rates between 0.1 and 100 s^−1^ and the shear stress rates between 0.1 and 30 Pa, at 25 °C. j) Photographs demonstrated the self‐healing behavior of Emb@CMCS‐OD/CMCS‐OD. Data were expressed as mean ± SD. *n* = 3.

Subsequently, The Emb was loaded into the CMCD‐OD. The color of CMCD‐OD changed from transparent and colorless to a uniform purplish‐red upon loading with Emb (**Figure** **3**a). SEM images displayed the microscopic changes in Emb@CMCS‐OD and CMCS‐OD in Figure 3b. Due to the higher porosity of the hydrogels and the good fusion relationship of Emb in the three‐dimensional network of hydrogels, no Emb crystalline particles caused by uneven distribution were observed. These indicated that Emb loading did not affect the hydrogel structure, and that the hydrogel system served as a good delivery vehicle for Emb.

Fourier‐transform infrared spectroscopy (FT‐IR) measurements (Figure 3c) demonstrated distinct absorption peaks corresponding to the vibrations of the C═N, C─N, and N─H bonds within the hydrogel at ≈1606, 1334, and 3312 cm^−1^. These absorption peaks illustrated the formation of Schiff base bonds in the hydrogels. CMCS‐OD was prepared by crosslinking CMCS and ODex via the Schiff base reaction. The Schiff base reaction is a nucleophilic addition reaction between an aldehyde and a primary amine, in which the nitrogen atom of the amine attacks the carbon atom of the carbonyl group, forming an *α*‐hydroxyamine, which then dehydrates to form an imine (C═N).^[^ ^32^ ^]^ During the crosslinking process, a C─N bond is formed between the carbon atom of ODex and the nitrogen atom of CMCS in the first nucleophilic addition step, whereas a C═N bond is formed in the second dehydration step.^[^ ^33^ ^]^ The corresponding characteristic peaks of these bonds were observed in the FT‐IR spectrum, thereby confirming the dual‐network structure of CMCS‐OD. This dynamic Schiff bond structure is associated with the self‐healing ability of CMCS‐OD.^[^ ^25^ ^]^ The FT‐IR analysis of Schiff base linkages was characterized by a broad absorption peak for N─H bond stretching vibrations in the range of 3250–3287 cm^−1^, and the presence of imine groups C═N stretching vibrations between 1603 and 1650 cm^−1^. The stretching vibrations of the C─N single bonds exhibited more dispersed characteristic absorption peaks, with specific positions varying depending on the structure and environment of the compound. Typically, the absorption peaks for C─N single bonds are located in the range of 1000–1250 cm^−1^, though this range is not absolute and must be evaluated in conjunction with the spectral information of the specific compound.^[^ ^34^ ^]^ These results corroborate the FT‐IR results.

When Emb was loaded into CMCS‐OD, it is noteworthy that the Schiff base reaction usually occurs in compounds containing active carbonyl groups, its participation in the Schiff base reaction might be low.^[^ ^35^ ^]^ The characteristic absorption peaks of Emb (767, 1381, 1621, and 2830 cm^−1^) were also detected in Emb@CMCS‐OD,^[^ ^14^ ^]^ and their intensities at 2830, 767, and 1381 cm^−1^ were attributed to the emergence of O─H and C═C bonds after Emb was incorporated into the hydrogel system. The disappearance of the small peak at 1621 cm^−1^ was assigned to the C═O stretching after Emb was integrated into the hydrogel system. These data indicated that CMCS‐OD successfully loaded Emb.

Moreover, the presence of carboxyl groups (‐COOH) within the hydrogel network was confirmed by the C═O, O─H, and C─O absorption peaks. This corresponds to the peaks observed near 1621, 2830, and 1050 cm^−1^ in the Emb@CMCS‐OD spectrum. Research has indicated that the characteristic absorption peaks of ─COOH typically include a broad band absorption for the O─H stretching vibration in the range of 3200–2500 cm^−1^, and a strong absorption peak for the C═O stretching vibration between 1710 and 1680 cm^−1^. Additionally, C─O stretching vibrations may result in absorption within the range of 1300–1210 cm^−1^. It can be inferred that the characteristic absorption peaks of the ─COOH bond fall within the aforementioned frequency ranges.^[^ ^36^ ^]^ The ─COOH groups play a pivotal role in enabling pH‐responsive drug release from CMCS‐OD.^[^ ^21^, ^22^, ^23^ ^]^

Figure S2 (Supporting Information) showed the NMR spectroscopic analysis of CMCS‐OD and Emb@CMCS‐OD comparing their structural differences through chemical shifts (horizontal axis) and signal intensities (vertical axis).^[^ ^37^ ^]^ The ^1^H‐NMR spectrum of CMCS‐OD displays peaks at 2.7 and 3.2 ppm, representing amino hydrogen (─NH_2_) and carboxylic hydrogen (─COOH), and indicates a representative structure. In contrast, the spectrum of Emb@CMCS‐OD showed significantly enhanced peaks at these chemical shifts, with intensities exceeding 900 arbitrary units (a.u.), and introduces new minor peaks at −0.23 and 1.33 ppm, likely corresponding to methyl (─CH_3_) hydrogens on the side chains of Emb.^[^ ^38^ ^]^ The enhancement of these peaks is associated with the aromatic hydrogen atoms (─C_6_H_4_─) on Emb, where the weak electron‐donating effect of the methyl groups on the benzene ring led to lower chemical shifts and higher peak intensities.^[^ ^38^ ^]^ The absence of these hydrogen nuclei signals in CMCS‐OD, owing to the lack of benzene ring structures, confirmed the successful incorporation of Emb.

Through X‐ray photoelectron spectroscopy (XPS) analysis, the O^1s^ spectrum in Figure S3 (Supporting Information) showed a higher peak intensity (531.33 eV for Emb@CMCS‐OD than for CMCS‐OD, indicating an increase in the hydroxyl (─OH) and carbonyl (─C═O) contents due to the addition of Emb. The C^1s^ spectrum revealed a lower peak intensity (284.80 eV for Emb@CMCS‐OD than for CMCS‐OD, thereby signifying a reduction in carbon–carbon (C–C) or carbon‐hydrogen (C─H) bonds, which was associated with the decrease in the C─C and C─H ratios attributable to the aromatic ring structure of Emb. The changes at 286 and 288 eV reflected an increase in C─O and C═O, respectively, which indicated that the introduction of Emb augmented the number of oxygen‐containing carbon compounds. The presence of carbonyl, methylene, and hydroxyl groups and aromatic rings in the structure of Emb led to an enhancement in the C─O and C═O bonds, thereby resulting in intensified XPS peak intensities.^[^ ^39^ ^]^ These findings provided conclusive evidence for the successful loading of Emb into the CMCS‐OD system.

After Emb was successfully loaded, the release profile of the hydrogel system was assessed. To quantify the concentration of Emb, a standard curve was created based on the correlation between the Emb concentration^[^ ^40^ ^]^ and absorption intensity (Figure S4a, Supporting Information). The molecular structure of Emb was demonstrated in Figure S4b (Supporting Information). Figure S5 (Supporting Information) showed a steep initial release from Emb@CMCS‐OD under different pH and temperature conditions, followed by a gradual decrease in the release rate after 12 h and stabilization at 24 h. After 48 h, the release rate plateaued and remained constant. By the 15th day, cumulative release ratio reached 90% and cumulative release mass nearly reached 1800 µg (Figure 3d,e). A similar burst pattern of Emb release under different pH and temperature conditions may be related to the rapid dissolution of Emb particles that were either adhered or incompletely loaded in the superficial layers of the hydrogel.^[^ ^41^ ^]^ This may play an essential role in reducing periodontal inflammation by rapidly increasing the local Emb concentration.

In an environment with a pH of 5.0, Emb released 15% of its total amount within the first 8 h. In contrast, at pH 7.4, only 10% of the Emb was released during the same period (Figure S5a, Supporting Information). This difference can be attributed to the pH‐responsive reaction of the Emb@CMCS‐OD delivery system. CMCS contains a large number of carboxyl groups (─COOH), which are ionized into negatively charged carboxylate ions (─COO─) under acidic conditions.^[^ ^21^, ^22^ ^]^ Simultaneously, the crosslinked CMCS‐OD network is composed of numerous amide bonds (─CONH─) that can react with hydrogen ions (H^+^) to produce cationic ‐NH3^+^. Greater amounts of ‐NH3^+^ are generated under more acidic conditions, which leads to an increase in the buffering capacity of the gel system.^[^ ^22^ ^]^ These features enable the network to loosen, which benefits the discharge of medication^[^ ^22^, ^42^ ^]^ and consequently clarifies the variation in the release curves at pH 5.0 and pH 7.4. At 37 °C, the ultimate release of Emb was ≈10%, higher than 25 °C (Figure 3e; Figure S5b, Supporting Information). This phenomenon was attributed to the increased solubility and diffusion coefficient of Emb at higher temperatures, which resulted in a higher quantity of the ultimate release. These results demonstrate the positive responsiveness of the CMCS‐OD delivery system to pH and temperature. Stimuli‐responsive delivery systems support on‐demand controlled‐release activities that can carry drugs more effectively to diseased tissues, maximize therapeutic effectiveness, and minimize the toxicity of delivered drugs to healthy tissues. Tissue inflammation is commonly followed by a slight increase in local temperature and a decrease in environmental pH.^[^ ^43^ ^]^ The slight temperature increase and weakly acidic pH in inflamed tissues are, therefore, regarded as important stimuli in the design of a smart drug delivery system.^[^ ^43^ ^]^ Consequently, properly designed drug carriers that can intelligently distinguish between diseased and healthy tissues are critical for improving treatment efficacy.^[^ ^42^ ^]^

The injectabilities of CMCS‐OD (colorless) and Emb@CMCS‐OD (purple‐red) were investigated. Them were filled separately into a two‐syringe system to form hydrogels immediately after injection to write “SYSU” in Figure 3f,g and Video S1 (Supporting Information).^[^ ^44^ ^]^ Furthermore, rheological performance tests showed that the viscosity of Emb@CMCS‐OD rapidly decreased with increasing shear rate/stress, thereby exhibiting shear‐thinning properties (Figure 3h,i).^[^ ^45^ ^]^ These results revealed the excellent injectability and convenience of filling irregular periodontal pockets.^[^ ^46^ ^]^

As shown in Figure 3j, this experiment aimed to explore the self‐healing capabilities of Emb‐loaded CMCS‐OD compared to unloaded CMCS‐OD, specifically their ability to automatically repair mechanical damage. CMCS‐OD (colorless) and Emb@CMCS‐OD (purple‐red) were cut in half, and upon close face‐to‐face contact with the cut surfaces, a clear self‐healing phenomenon was observed at the interface, where both types of hydrogels could remerge into a single entity. Even when a tensile force was applied to the healed hydrogels, causing them to stretch, the previously separated halves remained intact. The observed self‐healing results for both hydrogels were similar, showing no significant differences, indicating that both possess notable self‐healing capabilities and can effectively self‐repair within a short period. Considering that CMCS‐OD before loading Emb was colorless and transparent, to make the self‐healing interface more visually apparent, we dyed CMCS‐OD blue and magenta for the self‐healing experiments under different temperature conditions. We found the rise of temperature significantly enhanced the self‐healing process after the two colors of the CMCS‐OD were placed in contact at 37 and 25 °C, respectively (Figure S6, Supporting Information).

As mentioned above, FT‐IR, NMR, and XPS experiments showed that dynamic hydrogel networks were successfully formed between CMCS and OD via synergistic chemical and physical cross‐linking (Figure 3c; Figures S2 and S3, Supporting Information). The self‐healing mechanism of the hydrogel was attributed to the dynamic cleavage of imine and hydrogen bonds and the recombination of polymer networks, thereby indicating that self‐healing hydrogels uniquely provide a sustained protective treatment environment for periodontal lesions. Compared with traditional treatments, they can quickly restore their structure and function after physical damage, thereby maintaining the integrity of the treatment area and reducing the risk of secondary infections.^[^ ^47^ ^]^ They also ensured long‐term stability of the material in the periodontal pocket, enabling sustained and controlled drug release, which is particularly important for the long‐term treatment of chronic periodontitis.^[^ ^45^ ^]^ Additionally, this characteristic can reduce the frequency of medication administration, which is crucial for improving patient treatment experiences, especially for those who fear or feel uncomfortable with traditional dental treatments, potentially lowering the overall treatment costs.^[^ ^25^ ^]^

Overall, Emb@CMCS‐OD demonstrated distinct pH and temperature responsiveness during the release process. Its sustained‐release capability, injectability, and self‐healing properties facilitate convenient administration in clinical settings and extend the bioactivity of Emb at the site of the lesion.

### 2.2 Antibacterial Activity of Emb@CMCS‐OD

*P. gingivalis* is a central factor in periodontitis‐induced bone loss.^[^ ^48^ ^]^ The optimal antimicrobial concentration of Emb against *P. gingivalis* was determined based on the disc diffusion susceptibility test and live/dead bacterial staining. This examination demonstrated that there was no significant variance in antibacterial activity higher than concentrations of 250 µm (Figure S7, Supporting Information). As a consequence, a concentration at 250 µm of Emb was used for the succeeding experiments. Previous research has indicated that the synergistic application of Emb‐chitosan‐gold nanoparticles with ciprofloxacin exhibits inhibitory effects against multidrug‐resistant strains (*P. aeruginosa* resistant strains (PA‐r) and *E. coli* resistant strains (EC‐r)).^[^ ^14^ ^]^ Among the mechanisms of its antibacterial action, Emb demonstrated the potential to block efflux pumps (EPs) that maintain the osmotic balance between the interior and exterior of microbial membranes. The interaction between Emb and relevant efflux pump proteins was characterized by molecular docking to explore the possible binding modes between Emb and amino acid residues. These include efflux pump proteins from the PA‐r MexAB‐OprM (MexA) and EC‐r AcrAB‐TolC systems, suggesting that Emb can act on the active binding sites of proteins through hydrogen bonding. The inhibition of efflux pumps slows down the rate at which antimicrobial drugs are expelled by resistant bacteria, thereby increasing the concentration of ciprofloxacin within the bacterial cell, thereby affecting bacterial DNA gyrase/topoisomerase. This grants Emb its nanoformulation‐killing activity against multidrug‐resistant bacterial strains in the environment.^[^ ^14^ ^]^ Furthermore, the pathogenicity and biofilm formation of bacteria are closely related, with recent studies indicating that Emb disrupts the cell‐to‐cell communication mechanism used by gram‐negative pathogens to regulate gene expression in response to changes in cell population density; this is a process known as quorum sensing (QS). This leads to inhibition of virulence factor synthesis and biofilm formation. Emb inhibits QS‐dependent virulence factors and biofilm development, thereby impairing the ability of bacteria to coordinate attacks and defend themselves against antibiotics. This reduces their pathogenicity and enhances the efficacy of antimicrobial drugs.^[^ ^49^ ^]^ Therefore, we believe that this mechanism is one of the key reasons Emb exerts its antimicrobial effects against *P. gingivalis* at lower concentrations.

**Figure 4:**
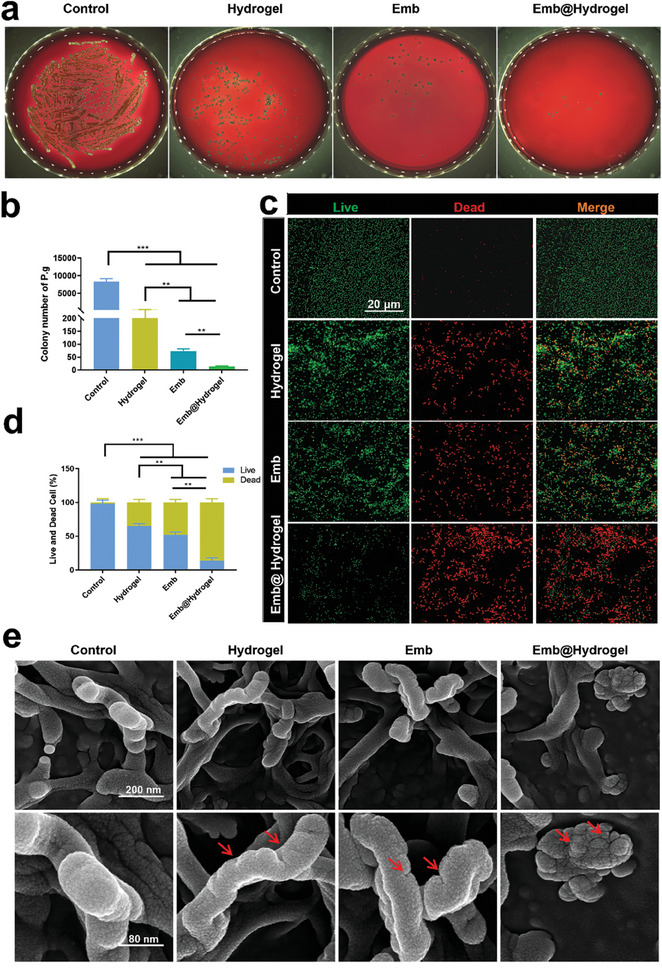
Antibacterial activity of Emb@CMCS‐OD on *P. gingivalis*. a,b) Colony formation of *P. gingivalis* on BHI plates. The images showed representative colonies and the histogram showed the quantitative analysis of the CFU. c,d) Live/dead staining of *P. gingivalis*. The images showed the fluorescence of live (green) and dead (red) bacteria and the histogram showed the quantitative analysis of the live/dead ratio. e) SEM images of *P. gingivalis* surface morphology. Data were expressed as mean ± SD. *n* = 3, *p < 0.05; **p < 0.01; ***p < 0.001.

As shown in **Figure** **4**a–d, the number of *P. gingivalis* in the brain‐heart infusion agar (BHI) medium and BHI blood plates was lower in the intervention group than in the control group. The bactericidal activity of CMCS‐OD may be linked to the cationic surface of the network, which disrupts bacterial integrity.^[^ ^50^ ^]^ Although hydrogel have been found to have antimicrobial properties, their effects are weaker than those of Emb. The antimicrobial properties of Emb@CMCS‐OD were significantly enhanced after the Emb was loaded onto the hydrogel. Similar to the original surface morphology of *P. gingivalis* in the control group, SEM observations revealed noticeable damage to the outer membrane structure of *P. gingivalis* in the Emb/Emb@CMCS‐OD group (Figure 4e). *P. gingivalis* was most visibly damaged by Emb@CMCS‐OD treatment and showed significant dehydration and shrinkage (Figure 4e). The improved antibacterial effect of Emb@CMCS‐OD on *P. gingivalis* was attributed to the synergistic effects of Emb and hydrogel system. The crosslinked network of the hydrogel facilitates the adhesion and disruption of *P. gingivalis*, which has been reported to trap bacteria via electrostatic attraction to the environment.^[^ ^51^, ^52^ ^]^ Furthermore, Emb maintained a high local concentration because of the sustained‐release effect of the CMCS‐OD delivery system. Under these conditions, Emb eliminated *P. gingivalis* directly and efficiently. This evidence confirms that Emb@CMCS‐OD has excellent antimicrobial properties.

### 2.3 Restoration of Immune Dysregulation and Osteogenesis by Emb@CMCS‐OD

The viabilities of mouse fibroblast cells (L929), periodontal ligament stem cells (PDLSCs), and mouse macrophage cells (RAW264.7) were determined using the CCK8 kit when they were co‐cultured with hydrogels at varying concentrations (25, 50, and 100 mg mL^−1^). These results indicated that the hydrogel at a concentration of 50 mg mL^−1^ did not compromise the viability of RAW264.7 cells. However, when the concentration was increased to 100 mg mL^−1^, the viability of RAW264.7% decreased to below 80% (Figure S8a, Supporting Information). Hydrogels at concentrations of 50 and 100 mg mL^−1^ demonstrated no negative effects on the viability of L929 cells and PDLSCs (Figure S8b,c, Supporting Information). Based on these outcomes, to avoid affecting the viability of RAW264.7, we opted for a hydrogel concentration of 50 mg mL^−1^ for subsequent experiments. Furthermore, we examined the effects of co‐culturing Emb with RAW264.7 cells in the range of 2.5–20 µm. The results revealed that Emb at a concentration of 5 µm exhibited the most notable anti‐inflammatory effect (Figure S9, Supporting Information). Hence, we utilized 5 µm Emb for further experiments.

As shown in the live/dead staining images, there was no significant difference in the cell proliferation rate and dead cell clumps of RAW264.7 among the different groups on days 2 and 4 (Figure S10a, Supporting Information). The live/dead staining for L929 cells were similar to RAW264.7 (Figure S11a, Supporting Information) and PDLSCs (Figure S12a, Supporting Information). The CCK‐8 test showed that the viability of RAW264.7 (Figure S10b, Supporting Information), L929 (Figure S11b, Supporting Information) and PDLSCs (Figure S12b, Supporting Information) in each group exceeded 95%. Notably, compared to other intervention groups co‐cultured with RAW264.7, the Emb@CMCS‐OD group exhibited slightly higher cell viability, which is similar to that observed in PDLSCs (Figures S10b and S12b, Supporting Information).

**Figure 5:**
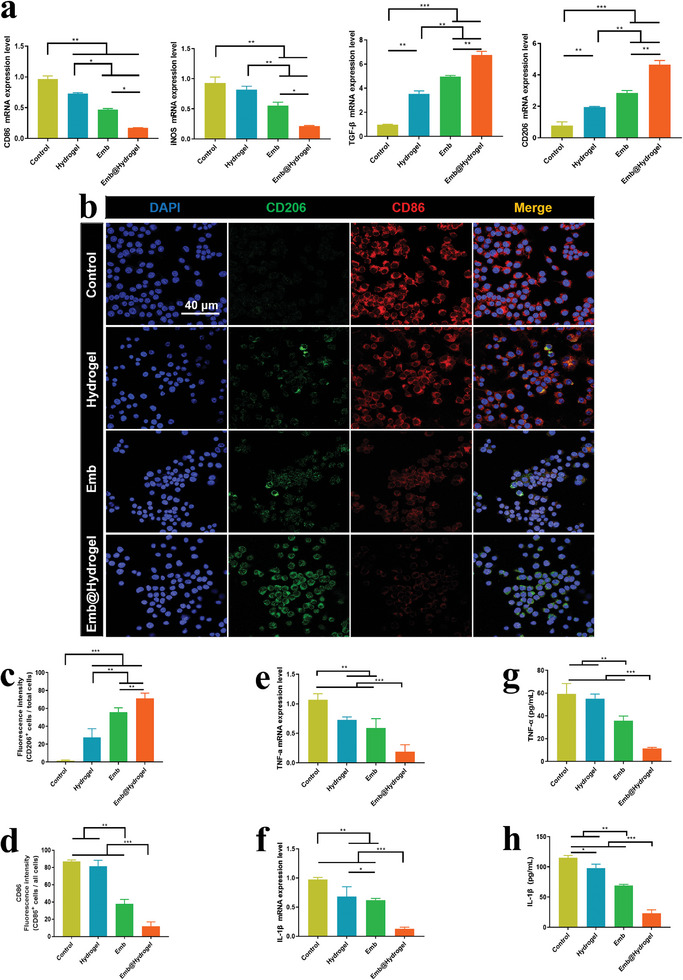
Anti‐inflammatory effects of Emb@CMCS‐OD on macrophages. a) qRT‐PCR analysis of the mRNA expression of M1 (*CD86* and *iNOS*) and M2 (*TGF‐β* and *CD206*) macrophage markers in macrophages. b–d) Immunofluorescence staining and quantitative analysis of the expression of M2 (CD206, green) and M1 (CD86, red) macrophage–phenotype markers in macrophages. e,f) qRT‐PCR analysis of the mRNA expression of pro‐inflammatory cytokines (*TNF‐α* and *IL‐1β*) in macrophages. g,h) ELISA analysis of the secretion of TNF‐*α* and IL‐1*β* in the supernatant of macrophages. Data were expressed as mean ± SD. *n* = 3, *p < 0.05; **p < 0.01; ***p < 0.001.

Considering a substantial role in M1‐M2 transformation of macrophages in regulating periodontitis, as previously mentioned.^[^ ^52^ ^]^ RAW264.7 cells were treated with LPS to mimic the inflammatory environment. As presented in **Figure** **5**a, compared with other groups, the Emb@CMCS‐OD remarkably upregulated the mRNA expression of M2 macrophage–phenotype markers (*CD206, TGF‐β*). In contrast, it downregulates the expression of M1 macrophagephenotype markers (*CD86, iNOS*). Simultaneously, it was observed that the ability of CMCS‐OD to promote M2 polarization of macrophages was greater than its ability to inhibit M1 polarization, which is consistent with previous research.^[^ ^53^ ^]^ Similarly, immunofluorescence analysis showed that the level of CD206 was dramatically increased, while that of CD86 was decreased in macrophages treated with Emb@CMCS‐OD (Figure 5b–d). We further validated the effects of Emb, CMCS‐OD, and Emb@CMCS‐OD on the polarization of RAW264.7 cells through flow cytometry. These results demonstrated that all treatments actively facilitated the transition of macrophages from the M1 inflammatory phenotype to the M2 anti‐inflammatory phenotype. Notably, Emb slightly outperformed Emb@CMCS‐OD in inhibiting the M1 phenotype (CD86^+^CD206^−^), whereas Emb@CMCS‐OD was most effective in promoting the M2 phenotype (CD86^−^CD206^+^). The reduction in the M1/M2 ratio further confirmed this phenotypic transition, thereby indicating that Emb@CMCS‐OD had a superior effect in promoting this transformation compared with the application of Emb or CMCS‐OD alone (Figure S13, Supporting Information).

Overall, Emb@CMCS‐OD is an efficient immunoregulatory strategy. Specifically, overabundance of the M1 subpopulation has been implicated in the initiation of an uncontrolled inflammatory cascade that eventually causes damage to the alveolar bone and supporting tissue.^[^ ^54^ ^]^ Conversely, M2 macrophages attract mesenchymal stem cells (MSCs) to affected areas, which can facilitate bone regeneration.^[^ ^55^, ^56^ ^]^ In short, addressing the macrophage polarization imbalance is a critical target in the treatment of bone destruction in periodontitis.

The expression of inflammatory cytokines was measured in macrophages. As showed in Figure 5e,f, it was found that Emb@CMCS‐OD extremely suppressed the mRNA expression of pro‐inflammatory cytokines including *TNF‐α* and *IL‐1β* compared with other groups. The CMCS‐OD and Emb groups also exhibited inhibitory effects on the expression of these cytokines. The superior anti‐inflammatory properties of the composite hydrogels were confirmed by ELISA (Figure 5g,h). Previous studies have indicated that CMCS can lower the levels of macrophage transcription factors pSTAT1/pSTAT6, suppress IL‐1*β* expression, and stimulate macrophage M2 phenotypic transformation, which align with our findings. The molecular mechanism of CMCS is directly associated with TLR4/MyD88/NF‐κB pathway activation.^[^ ^53^ ^]^

When combined with CMCS‐OD, Emb demonstrated exceptional performance, with remarkable therapeutic effects attributed to the following mechanisms: First, Emb effectively reduced the expression of pro‐inflammatory cytokines such as IL‐1*β* by inhibiting the NF‐κB pathway,^[^ ^15^ ^]^ and also blocked RANKL‐induced osteoclast differentiation,^[^ ^19^ ^]^ as validated in our experimental results (Figure S14a–d, Supporting Information). Recent studies have identified Emb as a highly selective GPR84 agonist.^[^ ^18^ ^]^ Activation of GPR84 in macrophages has been reported to participating in modulate cytokine secretion of macrophages via G12/13 and cAMP signaling pathway.^[^ ^18^ ^]^ These indicate that Emb may regulate macrophage polarization through GPR84 receptor. Furthermore, Emb decreases the expression of pro‐inflammatory cytokines in M1 macrophages while increasing the production of anti‐inflammatory cytokines, thereby demonstrating its dual role in alleviating inflammation and promoting tissue repair.^[^ ^16^, ^17^, ^18^, ^19^, ^20^ ^]^ The therapeutic action of Emb has been reported to positively impact LPS‐induced acute kidney injury primarily through the inhibition of NF‐κB pathway activation, thus, inspiring our application in periodontitis.^[^ ^16^ ^]^ Additionally, researchers have discovered that Emb exerts its protective effects mainly by upregulating the Nrf2/HO‐1 signaling pathway to enhance antioxidant defense, thereby mitigating kidney toxicity induced by cisplatin.^[^ ^57^ ^]^ Emb, an inhibitor of X‐linked apoptotic proteins, directly reduces the expression of XIAP in gingival epithelial cells (GECs).^[^ ^58^ ^]^ XIAP is a protein that inhibits apoptosis, and its overexpression can hinder the normal death of infected cells, leading to persistent infection and survival of pathogens, such as *P. gingivalis*.^[^ ^59^ ^]^ By decreasing XIAP expression, Emb aids in restoring the apoptotic response of cells to infection, thereby reducing the survival niche for pathogens. The aforementioned studies, along with our experimental results, provide a preliminary mechanistic explanation of the role of Emb in addressing inflammation and treating periodontitis.

Since the effect of Emb on osteogenic activity has not yet been elucidated, we investigated the effects of Emb@CMCS‐OD, CMCS‐OD, and Emb on the osteogenic differentiation of PDLSCs under LPS‐induced inflammatory conditions and found that the transcript levels of osteogenic factors, including alkaline phosphatase (ALP) and osteocalcin (OCN), were significantly downregulated in PDLSCs (Figure S15, Supporting Information) in a chronic inflammatory microenvironment. Similarly, in the ALP and ARS staining results, ALP activity was reduced in PDLSCs under inflammatory conditions. LPS inhibited the formation of mineralized nodules in PDLSCs (Figure S15a,b, Supporting Information). The application of Emb@CMCS‐ODs to PDLSCs accelerated osteogenesis. In the stimulated inflammatory PDLSCs groups (LPS + Emb@CMCS‐OD), the mRNA levels of *ALP* and *OCN* levels (Figure S15c, Supporting Information) were elevated. In particular, the mRNA levels of *OCN* changed significantly compared to those of *ALP*.

In addition, ALP and ARS staining showed higher levels of ALP and calcium deposition when Emb@CMCS‐OD was used for inflammatory PDLSCs (Figure S15a,b, Supporting Information). Moreover, ALP and ARS staining also showed a weaker increase in the levels of ALP and calcium deposition when Emb and CMCS‐OD were used in inflammatory PDLSCs than when Emb@CMCS‐OD was used (Figure S15a,b, Supporting Information). In contrast to the control groups (LPS), although Emb and CMCS‐OD inhibited *ALP* and *OCN* mRNA expression in PDLSCs in an inflammatory microenvironment, the inhibitory effect of Emb@CMCS‐OD was more pronounced than the combined effects of Emb and CMCS‐OD (Figure S15c, Supporting Information).

Meanwhile, CMCS‐OD has an internal hydrophobic cavity and an external hydrophilic surface. The internal hydrophobicity is beneficial for Emb molecule encapsulation, whereas the external hydrophilic surface enhances the hydrophilicity of the hydrophobic compound.^[^ ^60^ ^]^ This mechanism improved the solubility of Emb in the co‐culture system and enhanced its biological activity.^[^ ^61^, ^62^, ^63^ ^]^ Based on the above data, we found that Emb@CMCS‐OD effectively improved osteogenic activity and reduced osteoclastic activity, which indicates that Emb@CMCS‐OD has great potential for periodontal regeneration in vitro.

This proved that the well‐designed Emb@CMCS‐OD efficiently suppressed inflammation and restored immune dysregulation and osteogenesis.

### 2.4 Therapeutic Effect of Emb@CMCS‐OD in Rat Periodontitis Model

**Figure 6:**
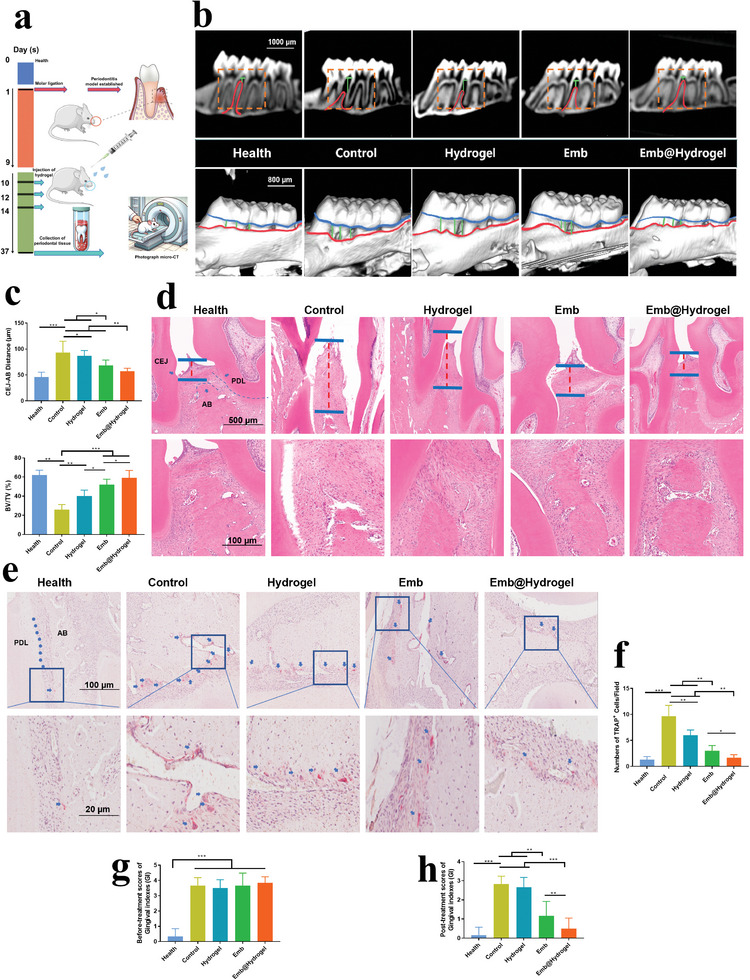
Emb@CMCS‐OD enhanced alveolar bone regeneration in a ligation‐induced periodontitis model of rats. a) Schematic illustration of the establishment and operation of the rat periodontitis model. b,c) Micro‐CT images (cross‐sectional and 3D reconstructed views) and quantitative analysis of the CEJ‐AB distance and BV/TV ratio. d) H&E staining of the alveolar bone sections. e) TRAP staining of the alveolar bone sections. f) Quantitative analysis of the TRAP^+^ cells per bone area. g,h) Gingival index (GI) scores of the rats before and after treatment. Data are expressed as mean ± SD, *n* = 6. *p < 0.05; **p < 0.01; ***p < 0.001.

The process, including the establishment of a ligation‐induced periodontitis model,^[^ ^64^ ^]^ Emb@CMCS‐OD administration, sample collection, and examination, is presented in **Figures** **6**a and S16a–d (Supporting Information). Micro‐CT analysis revealed that compared to the healthy group, the control group exhibited significant vertical bone loss in the alveolar bone. The distance between the cemento‐enamel junction and alveolar bone crest (CEJ‐AB) notably increased, thereby confirming the successful establishment of the periodontitis model. Rats treated with either Emb or CMCS‐OD alone showed improved alveolar bone recovery. Nonetheless, the recovery of the alveolar bone was more striking when treated with Emb@CMCS‐OD, with a recovery level almost identical to that of healthy alveolar bone (Figure 6b,c). According to the bone volume/total volume (BV/TV) ratio, the health group exhibited the highest BV/TV values, reflecting the most intact alveolar bone structure. The control group that received no treatment had the lowest BV/TV values, which indicates a significant reduction in alveolar bone mass following periodontitis. After individual treatments with the hydrogel and Embs, there was an improvement in BV/TV values, but they remained significantly lower than normal levels. This suggests that these single treatment modalities have limited effects on the regeneration of alveolar bone. Meanwhile, the BV/TV value of the Emb@Hydrogel combined treatment group was the closest to that of the health group (Figure 6c). The BV/TV value was consistent with the results of 3D reconstruction images, thereby confirming that the combined treatment regimen had a significant advantage in addressing alveolar bone destruction.

H&E staining indicated that the periodontium of the control group exhibited significant infiltration of inflammation, along with a loosely junctional epithelium and disorganized fiber tissue structure. The alveolar ridge apex exhibits considerable resorption. The periodontal tissues in the Emb@CMCS‐OD group demonstrated exceptional restoration of junctional epithelium connections, marked by a reduction in inflammatory cell infiltration. The arrangement of the periodontal ligament (PDL) tended to be normal, and nearly recovered the CEJ‐AB distance to that of healthy rats (Figure 6d). Emb had a therapeutic effect, and the CMCS‐OD group had a weaker effect. In addition, compared with health organs, no significant abnormalities were observed in the intervention groups, thereby suggesting that subsequent degradation in vivo did not cause apparent systemic side effects (Figure S17, Supporting Information). These findings demonstrated the extraordinary biocompatibility of Emb@CMCS‐OD.

TRAP staining was used to evaluate the number and activity of osteoclasts. As shown in Figure 6e,f, the control group exhibited a higher number of TRAP‐positive cells, with discernible red staining. In contrast, the group treated with Emb@CMCS‐OD showed a remarkable reduction in TRAP‐positive cells, which was lower than that in the Emb or hydrogel groups (Figure 6f). Collectively, the composite hydrogel was proven to be efficacious in periodontitis treatment, including the reduction of bone loss and promotion of bone regeneration.

It is noteworthy that gingival crevicular fluid was collected and measured from healthy rats and periodontitis rats. The results demonstrated that the pH of the periodontitis rats was lower than the healthy group of rats (Figure S18, Supporting Information). Gingival Index (GI) is a crucial clinical indicator for assessing the state of local periodontal inflammation.^[^ ^65^ ^]^ The effectiveness of treatments across different groups can be evaluated by the difference in bar graph results between the GI before (Figure 6g) and after treatment (Figure 6h). Thus, Emb@CMCS‐OD showed superior efficacy in alleviating local periodontal inflammation, especially in reducing post‐probing bleeding in the periodontal tissues.

**Figure 7:**
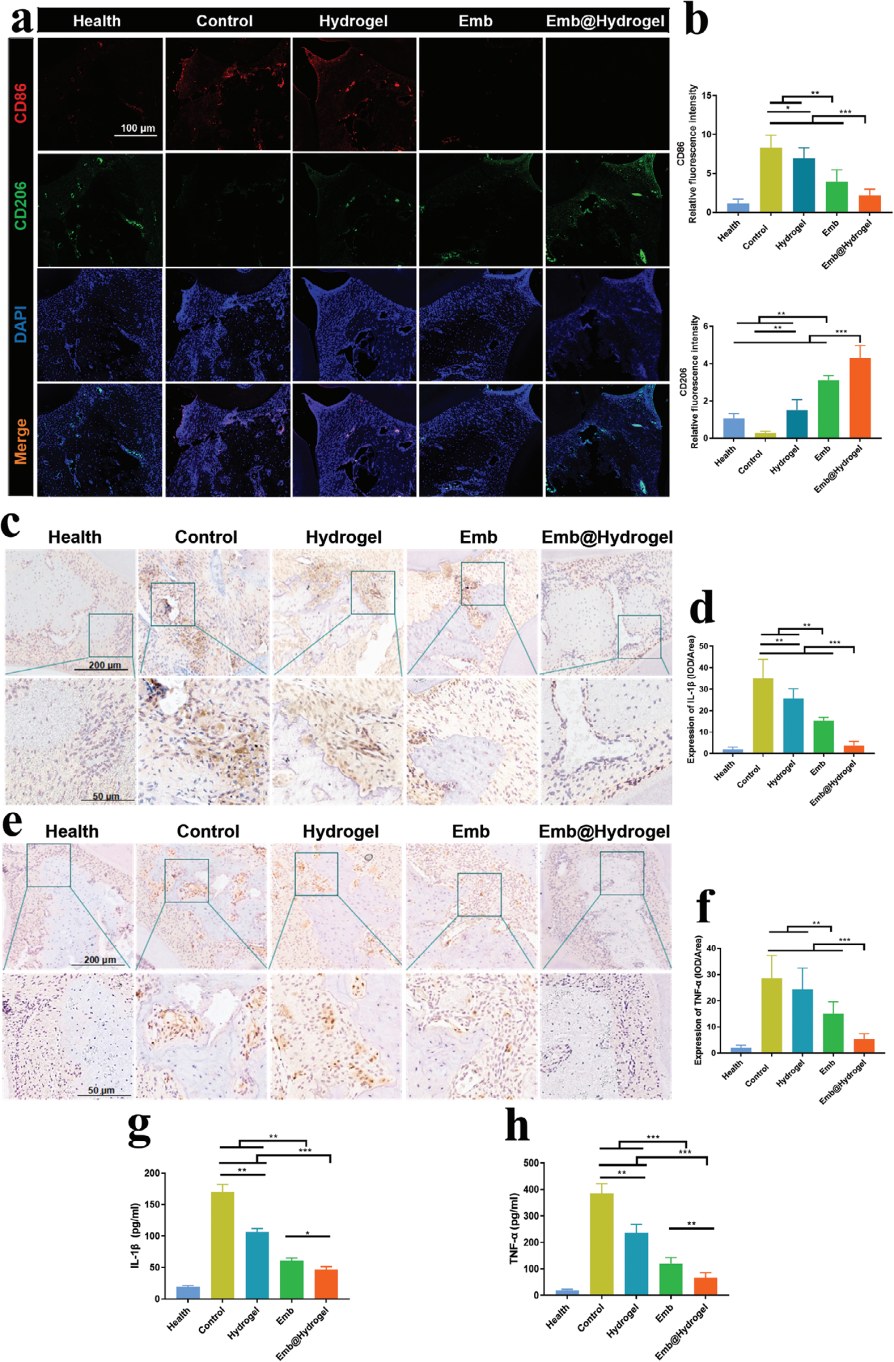
Histological evaluation of immunomodulatory of Emb@CMCS‐OD in a ligation‐induced periodontitis model of rats. a) Immunofluorescence staining of the periodontal tissues, showed the distribution of CD206^+^ cells (green) and CD86^+^ cells (red). b) Quantitative analysis of immunofluorescence images (CD206 and CD86). c) Immunohistochemical staining of the periodontal tissues, showed the expression of IL‐1*β*. d) Quantitative analysis of immunohistochemical staining (IL‐1*β*). e) Immunohistochemical staining of the periodontal tissues, showed the expression of TNF‐*α*. f) Quantitative analysis of immunohistochemical staining (TNF‐*α*). g,h) ELISA analysis of the periodontal tissues, showed the concentration of IL‐1*β* and TNF‐*α*. Data were expressed as mean ± SD, *n* = 6. *p < 0.05; **p < 0.01; ***p < 0.001.

Immunofluorescence staining and quantitative analysis (**Figure** **7**a,b) showed a decline in M1‐type macrophages and an increase in M2‐type macrophages in the Emb@CMCS‐OD group, which echoed the aforementioned results in vitro experiments. Furthermore, immunohistochemical staining revealed that positive staining for IL‐1*β* (Figure 7c,d) and TNF‐*α* (Figure 7e,f) in the periodontal tissues of the Emb@CMCS‐OD group were notably decreased compared to the control group.

Emb@CMCS‐OD group had significantly more OCN (osteogenesis marker, brown area) than the Control, indicated the osteogenic capacity of the Emb@CMCS‐OD was the best (Figure S19, Supporting Information). Besides, ELISA analysis of IL‐1*β* and TNF‐*α* levels in rat periodontal tissues again verified the outstanding anti‐inflammatory capacity of Emb@CMCS‐OD (Figure 7g,h).

Accordingly, the therapeutic effect of Emb@CMCS‐OD was primarily ascribed to the immunomodulation triggered by both the hydrogel and Emb, which prevented bone destruction caused by inflammation. Emb prevents RANKL‐induced osteoclast differentiation of RAW264.7.^[^ ^19^ ^]^ This may offer another explanation for the prevention of bone loss and alleviation of periodontitis.

Currently, minocycline hydrochloride, endowed with anti‐bacterial and anti‐collagenase activity, can be used to relieve clinical symptoms of periodontitis.^[^ ^66^ ^]^ Importantly, in addition to antibacterial activity, Emb can modulate osteo‐immune microenvironment to improve periodontitis. The combination of CMCS‐OD and Emb enable sustained release and efficiency of Emb, these overcomes frequent administration of drug. Collectively, the multifunctional hydrogel encapsulated hydrophobic Emb through its dual‐network structure to protect Emb from degradation by enzymes in the oral environment while improving its solubility. Additionally, the injectability of Emb@CMCS‐OD overcame the inability to fill irregular periodontal defects in conventional bone‐grafting surgeries, avoiding the formation of dead spaces and the risk of infection, and consequently optimizing the efficacy of bone regeneration.

Despite the self‐healing capability of the composite hydrogel, which allows for regeneration after fragmentation, hardness deficiency^[^ ^67^ ^]^ hinders the hydrogel from effectively maintaining space. Effective space maintenance correlates with the success rate of bone regeneration, in line with the “PASS” principle.^[^ ^68^ ^]^ We aim to strengthen the hardness of the hydrogels in subsequent studies. Thus, Emb@CMCS‐OD can cope with the complex oral microenvironment and suppress inflammation, thereby resulting in the superb restoration of periodontal bone loss.

## 3 Conclusion

A multifunctional injectable hydrogel system based on a dual dynamic network of ligands and Schiff base bonds was successfully prepared. The CMCS‐OD delivery system demonstrated pH‐responsive and self‐healing capabilities, enabling the flexible controlled release of Emb, thereby exhibiting potent antibacterial properties and the ability to reverse immune dysregulation. In particular, our observations revealed that Emb@CMCS‐OD coordinated the periodontitis microenvironment by inhibiting *P. gingivalis*, modulating the macrophage phenotype, and degrading inflammatory cytokines, ultimately contributing to alveolar bone regeneration. Specifically, Emb@CMCS‐OD improved the reprogramming of inflammatory macrophages into anti‐inflammatory cells while suppressing its secretion of TNF‐*α* and IL‐1*β* to exert anti‐inflammatory effects. Interestingly, Emb@CMCS‐OD not only inhibited osteoclast differentiation but also restored the osteogenesis function of PDLSCs, which might also contribute to the alleviation of bone loss in periodontitis. In summary, the antibacterial and immune coordination achieved by Emb@CMCS‐OD represent a convenient and efficient strategy for the clinical treatment of periodontitis.

## 4 Experimental Section

A detailed experimental section is provided in Supporting Information.

## Conflict of Interest

The authors declare no conflict of interest.

## Supporting information

Supporting Information

Supplemental Video 1

## Data Availability

The data that support the findings of this study are available from the corresponding author upon reasonable request.
