# Supplementary material for: A Microenvironment‐Responsive, Controlled Release Hydrogel Delivering Embelin to Promote Bone Repair of Periodontitis via Anti‐Infection and Osteo‐Immune Modulation
Source: Adv Sci (Weinh). 2024 Jul 8;11(34):2403786. doi: 10.1002/advs.202403786 (PMC11425865; doi:10.1002/advs.202403786)
Supplement: Supplementary file 1 — Supporting Information [file ADVS-11-2403786-s002.docx]

Supporting Information

**A Microenvironment-Responsive, Controlled Release Hydrogel Delivering Embelin to Promote Bone Repair of Periodontitis via Anti-infection and Osteo-immune Modulation**

*Guanming Cai^#^, Lin Ren^#^, Jiali Yu, Siqi Jiang, Gen Liu, Shujie Wu, Bin Cheng*, Weichang Li*, Juan Xia**

**Materials and Methods**

**1. Characterization of CMCS-OD**

**1.1 The Cross Linking Reactions of CMCS-OD**

The Schiff base reaction is a nucleophilic addition reaction between an aldehyde or a ketone and a primary amine, in which the nitrogen atom of the amine attacks the carbon atom of the carbonyl group, forming an α-hydroxyamine, which then dehydrates to form an imine (C=N). In the crosslinking process, the C-N bond was formed between the carbon atom of ODex and the nitrogen atom of CMCS in the first nucleophilic addition step; while the C=N bond was formed in the second dehydration step. The N-H bond was a covalent bond between the nitrogen and hydrogen atoms, present in the imine compounds.^[1-2]^

**1.2 Scanning Electron Microscopy (SEM)**

Hydrogel samples were lyophilized at room temperature for 24 h to remove surface moisture. The samples were then sputter-coated with a 10 nm gold layer to enhance electron conductivity. The samples were mounted on Scanning Electron Microscope (ZEISS SEM Sigma 360) stubs, and the SEM was operated at an accelerating voltage of 10 kV and a working distance of 10 mm. Images were taken at various magnifications to observe the microstructure of the hydrogels. The SEM software was used for preliminary analysis of pore structure and surface morphology.

**1.3 Roughness Parameters**

Hydrogel samples were cut into 1 cm x 1 cm squares and mounted on microscope slides. The Confocal Laser Scanning Microscope (ZEISS LSM 980) was set to an appropriate laser wavelength and focus was adjusted to capture the surface of the samples. XYZ scanning was performed to collect data on surface roughness. The microscope software was used to calculate roughness parameters like Ra and Rz.

**1.4 Porosity Assessment**

Hydrogel samples were lyophilized to preserve their microstructure. The lyophilized hydrogels were then imaged under SEM to observe their microstructure, including pores. Image analysis was conducted by randomly selecting five areas to measure pore size, distribution, and total porosity. The average values were calculated.

**1.5 Swelling Ratio Assessment**

The initial dry weight (W_dry) of the lyophilized hydrogel samples was recorded. The samples were then immersed in PBS at pH 7.4 and incubated at 37°C. At specific time points, the samples were removed, and surface water was gently blotted with filter paper. The wet weight (W_wet) was measured. The swelling ratio was calculated using the formula: Swelling ratio (%) = [(W_wet - W_dry) / W_dry] x 100%. The experiment was repeated at least three times to ensure reproducibility.

**1.6 Degradation Experiment**

For the in vitro degradation test, hydrogel samples of equal initial weight were soaked in PBS containing 1U/mL collagenase type I and shaken at 100 rpm/minute at room temperature. On the predetermined days, hydrogels were removed, lyophilized, and weighed. The remaining weight (%) of the hydrogels was calculated using the following equation: Weight of remaining hydrogel (%) = Wt / W1 × 100%, where W1 is the initial weight of the dry hydrogel and Wt is the weight of the remaining lyophilized hydrogel after degradation at different time points.

**1.7 Thermogravimetric Analysis (TGA)**

Approximately 5-10 mg of hydrogel sample was weighed and placed in the TGA instrument (TA Instruments Q500). The temperature was ramped from room temperature to 600°C at a rate of 10°C min^-1^. The mass change of the sample was recorded throughout the heating process. The TGA software was used to analyze the data and determine the thermal stability of the hydrogels, such as the temperature points of mass loss. The thermal decomposition stability of the hydrogels was analyzed based on the mass loss curve. The experiment was repeated at least three times for reproducibility.

**1.8 Rheological Performance Evaluation**

Precursor solutions were prepared and maintained at room temperature. A specific amount of the precursor solution was placed between the plates of the Rheometer (Malvern Kinexus Pro). The rheometer was set to a temperature of 37°C to simulate body temperature. A small amplitude oscillatory shear stress was applied to monitor changes in the storage modulus (G') and loss modulus (G'') during gelation. The time point when G' exceeded G'' and remained stable was recorded as the gelation time. Rheological curves during gel formation were analyzed to assess the gelation kinetics of the hydrogels.

**1.9 The Shear-Thinning Properties (Injectability) of CMCS-OD**

Before testing, samples were eliminated any stresses induced during processing. Test parameters included: shear rate range from 0.1 s⁻¹ gradually increasing to 100 s⁻¹; temperature: the entire test was conducted at a constant temperature of 25°C. Data collection and analysis: Viscosity measurements and shear stress recording were collected at each shear rate to record the rheological response.

**1.10 Hydrogel Self-Healing Capability Assessment**

Cut the differently stained hydrogel samples in half, making sure the cuts are clean. Bring the cut surfaces of the two halves into close contact. Maintain contact under pressure to promote the healing process. Afterwards, the self-healing situation of the incision was photographed and observed, and the morphological changes before and after repair were recorded. Repeat the experiment to verify the reproducibility of the self-healing ability.

**1.11 Fourier Transform infrared spectroscopy (FTIR) of Hydrogel**

The hydrogel samples were lyophilized to constant weight and ground into powder. Samples were prepared using the KBr pelletizing technique. Set the scanning range to 4000-400 cm^-1^ and the resolution to 4 cm^-1^. Continuous scans were performed to record the infrared absorption spectrum of the sample. Finally, spectral analysis software is used to identify and analyze characteristic absorption peaks to determine the chemical composition of the hydrogel.

**1.12 The Embelin Release Curve of Emb@CMCS-OD**

Prepare serial dilutions of Embelin standard solutions to construct a calibration curve. Take the Embelin-loaded hydrogel sample and put it into the dialysis bag, and then immerse it in pure water under different conditions (37°C/25°C or pH=5.0/7.4). Periodically remove a certain volume of release medium and replenish it with an equal volume of pure water. The absorbance of the release medium was measured using a UV-visible spectrophotometer (Shimadzu UV-1800). Calculate the Embelin concentration at each time point based on the calibration curve, and draw the Embelin release curve. Finally, the Embelin release data were analyzed to determine the medication loading capacity and release kinetic parameters of the hydrogels.

**1.13 Nuclear Magnetic Resonance(NMR) Spectroscopy Measurements**

The Embelin loaded in CMCS-OD was proved by proton nuclear magnetic resonance (^1^H NMR). CMCS-OD and Emb@CMCS-OD samples were dissolved in deuterium oxide to get ^1^H NMR spectra respectively. NMR were performed on Bruker Avance 300 MHz and Bruker Avance 400 MHz instruments by using a 90 degree pulse and a repetition time as to allow the magnetization to relax completely. The deuterated solvent for lock purpose was purchased from Sigma Aldrich (Milano, Italy).^[3]^

**1.14 X-ray photoelectron spectroscopy (XPS)**

The CMCS-OD and Emb@CMCS-OD were analyzed by X-ray photoelectron spectroscopy (XPS, PHI VersaProbe II, Physical Electronics Inc.). Hydrogel samples were lyophilized at room temperature for 24 h to remove surface moisture and prior to performing XPS. The XPS spectra were collected using monochromatic Al Kα X-ray source. ^[4]^

**2. Antibacterial Activity Assessment of Emb@CMCS-OD**

The *in vitro* antibacterial activity of each group was evaluated using the key periodontitis pathogen *Porphyromonas gingivalis* (*P. gingivalis*, ATCC33277). Initially, six groups (Control, 50 μM, 150 μM, 250 μM, 350 μM, 450 μM) were used for live/dead cell staining and antibacterial disk diffusion test to explore the antibacterial efficacy of Embelin. The following four groups were used: blank control group, that is, untreated bacteria, labeled as "Control"; CMCS-OD group, marked as "Hydrogel"; pure Embelin group, Embelin dose as same as in Emb@CMCS-OD, marked as “Emb”; and the Emb@CMCS-OD group, marked as “Emb@Hydrogel”.

**2.1 Disk Diffusion Susceptibility Test**

*P. gingivalis* was cultured to mid-log phase with an optical density of about 1.0 (OD = 1.0). 100 μL of *P. gingivalis* bacterial solution was inoculated on BHI blood plates as control and after absorption of the bacterial liquid, 6 mm diameter filter papers containing different concentrations of Embelin (0-450 μM) were placed on the medium surface, incubated at 37°C under strict anaerobic conditions for 48 h. After incubation, the diameters of the inhibition zones were measured with a protractor. The antibacterial ability of the materials was judged based on the size of the inhibition zones.

**2.2 Anti-bacterial activity in solid medium**

*P. gingivalis* was cultured to mid-log phase with an optical density of about 1.0 (OD = 1.0). The three interventions (Emb, CMCS-OD, and Emb@CMCS-OD ) were added to *P. gingivalis* culture and incubated for 2 h. Subsequently, 100 μL of *P. gingivalis* culture from each group was inoculated on BHI blood plates. Bacteria from the control group were spread directly without any intervention. Cultures were incubated at 37°C under strict anaerobic conditions for 48 h, and the total colony count of each group was recorded.

**2.3 Live/Dead Bacterial Staining**

*P. gingivalis* was cultured as mentioned. The three interventions as above were added to the *P. gingivalis* culture and incubated for 2 h. The control group used regular BHI liquid medium. All groups were cultured at 37°C for 48 h and then assessed using live/dead bacterial staining (LIVE/DEAD™ BacLight™ Bacterial Viability Kit, Thermo Fisher Scientific Scientific). Alive and dead bacteria were stained green and red, respectively.

**2.4 *In Situ* Antibacterial Activity**

*P. gingivalis* was cultured as mentioned. In the Hydrogel/Emb@Hydrogel group, 1mL CMCS-OD/Emb@CMCS-OD were injected into agar sheep blood culture medium pre-inoculated with *P. gingivalis*. For the Emb group, 1mL Embelin at a concentration of 250 μM was added onto agar sheep blood culture medium pre-inoculated with *P. gingivalis*. The *P. gingivalis* were pre-inoculated onto solidified agar sheep blood medium without any intervention as control. After incubating the above systems continuously at 37°C for 48 h. Furthermore, after ethanol gradient dehydration and low-temperature freeze-drying, the surface morphology was examined by SEM.

**3. Biocompatibility Assessment of Emb@CMCS-OD**

**3.1 Cell Grouping and Culture Conditions**

Mouse fibroblast cells (L929), Periodontal ligament stem cells (PDLSCs) and mouse macrophage cells (RAW264.7) were purchased from the Cell Bank of the Chinese Academy of Sciences.

RAW264.7, PDLSCs and L929 cells were cultured in Dulbecco's Modified Eagle Medium (DMEM, Gibco) containing 10% (v/v) fetal bovine serum (FBS, Sigma) and 1% penicillin-streptomycin (P/S), at 37°C in a 5% CO_2,_ 100% humidity, incubator with medium changed every 48 h. Cells from passages 3-5 were used for experiments.

**3.2 Cell Counting Kit-8 (CCK-8)**

L929, PDLSCs and Raw264.7 was used to evaluate the cell compatibility of CMCS-OD. In medium as mentioned with different concentrations of CMCS-OD (25, 50, 100 mg mL^-1^) were prepared. Cells above were seeded in 96-well plates at a density of 5×10^3^ for 24 h for adherence. Then cells were treated with the aforementioned medium for 24 h. Wells without any CMCS-OD served as blank controls. The operation was then performed according to the stand The Cell Counting Kit-8 (CCK-8; Dojindo Laboratories, Kumamoto, Japan). Based on preliminary experimental results, we selected a CMCS-OD culture medium concentration of 50 mg mL^-1^ and 5 μM Embelin for subsequent experiments.

**3.3 Live/Dead Cell Staining**

The cells culture scheme was the same as above. Live/dead cell staining was then performed according to the standard Thermo Fisher Scientific protocol (LIVE/DEAD Viability/Cytotoxicity Kit, L3224), and cells were observed and photographed under a fluorescence microscope (Leica, Germany). The effect of CMCS-OD on cell proliferation was assessed by observing live (green) and dead (red) cells.

Based on preliminary experimental results, we selected a CMCS-OD culture medium concentration of 50 mg mL^-1^ and 5 μM Embelin for subsequent experiments.

**3.4 *In Vivo* Biocompatibility Experiments**

Equal volumes of the interventions were injected into the gingival sulcus of maxillary second molar in SD rats in each group (buccal mesial, buccal central, buccal distal, palatal mesial, palatal central, palatal distal, a total of 200 μL at 6 sites). After 4 weeks, their major organs (heart, liver, spleen, lung, kidney) were obtained to explore pathological changes (*n* = 6). The animal experiments followed protocols approved by Institutional Animal Care and Use Committee (IACUC) of Sun Yat-Sen University (SYSU-IACUC-2022-002367) and conformed to the NIH Guide for the Care and Use of Laboratory Animals (NIH Publication No. 8023, revised 1978).

**4. *In Vitro* Anti-Inflammatory Activity Assessment of Emb@CMCS-OD**

All groups incubated with 1 µg mL^-1^ LPS. The negative control group marked as "Control". Cells were treated with CMCS-OD and LPS, marked as "Hydrogel". Cells were treated with LPS and pure Embelin at the same dosage as in Emb@CMCS-OD, marked as "Emb". The Emb@Hydrogel group treated with Emb@CMCS-OD and LPS, marked as "Emb@Hydrogel". Preliminary experiments (Figure S9a-g) indicated that the optimal anti-inflammatory effect of Embelin was at a concentration of 5μM, which was used for the experiments.

**4.1 Quantitative Real-Time Polymerase Chain Reaction (q-RT-PCR)**

RAW264.7 cells were seeded at a density of 1×10^5^ in 6-well plates until cell adherence. Cell grouping, interventions, and culture conditions were the same as before.

After co-culturing the interventions with cells for 24 h, total RNA from macrophages was extracted using TRIZOL (Life Technologies, Carlsbad, California, USA), according to the manufacturer's instructions, and cDNA was prepared by reverse transcription using the PrimeScript RT reagent kit (Takara, Kusatsu, Shiga, Japan). The gene expression of M1 phenotype markers (*CD86, iNOS*) and M2 phenotype markers (*CD206, TGF-β*), as well as periodontitis-related inflammatory factors: tumor necrosis factor α (*TNF-α*), and interleukin 1β (*IL-1β*), were detected by applying the SYBR Premix Ex Taq II kit (Takara). The housekeeping gene β-actin was used as an internal control. The mixture was loaded into a 96-well plate, and the change in fluorescence signal was monitored in real-time by a real-time fluorescence quantitative PCR instrument (The LightCycler® 96 Instrument, F. Hoffmann-La Roche Ltd, USA). Target gene expression levels were analyzed using the relative quantification method (The ΔΔCt method). Three replicates were set for each group. Primer sequences for Q-RT-PCR are shown in Table S2.

**4.2 Immunofluorescence Staining**

Cell grouping, interventions, and culture conditions were the same as before. After co-culturing the interventions with cells for 24 h, cells were washed with staining buffer (2% PBS), fixed with 4% paraformaldehyde, and treated with a permeabilizing agent (0.1% Triton X-100) to increase membrane permeability. Buffer containing bovine serum albumin (BSA) was typically used to block non-specific binding sites. Cells were incubated with CD86 (1:500, BioLegend, USA) and CD206 (1:500, Abcam, Cambridge, UK) antibodies overnight at 4°C. After washing with PBS, cells were incubated with Goat Anti-Rabbit IgG H&L (1:1000, Alexa Fluor® 488) and Goat Anti-Mouse IgG H&L (1:1000, Alexa Fluor® 647) at room temperature for 1 h, and stained with DAPI (Solarbio, China) for 5 minutes.

Fluorescence was detected using a ZEISS LSM 980 Confocal Laser Scanning Microscope. Five fields of view were randomly selected, and images were analyzed by FlowJo v.10 Software TreeStar Inc.

**4.3 Enzyme-linked Immunosorbent Assay (ELISA)**

Cell grouping, interventions, and culture conditions were the same as before. After co-culturing the interventions with cells for 24 h, supernatants were collected by centrifugation and the levels of tumor necrosis factor α (TNF-*α*) and interleukin 1β (IL-1*β*) in the supernatants were measured using ELISA kits (Thermo Fisher Scientific Scientific). The absorbance of each well was determined using an ELISA Reader (Thermo Multiskan MK3), with three replicates set for each group. The concentration of inflammatory factors in the samples was calculated based on the standard curve.

**4.4 Tartrate Resistant Acid Phosphatase (TRAP) Staining**

Cell grouping and culture conditions were the same as before, with cells seeded in six-well plates. In addition to RANKL (50ng mL^-1^), no other interventions were applied to the control group. The RANKL(50ng mL^-1^) + Emb (5 μM) and RANKL(50ng mL^-1^) + Emb (10 μM) were added to the two intervention groups respectively. After removing the medium, 3 mL of PBS was added gently to wash the cells. After removing the added PBS, 500 μL of precooled fixative was slowly added to avoid cell disruption, and left on ice for 10 minutes. Perform experimental operations according to TRAP staining kit. Under a light microscope, TRAP-positive cells are wine-red, and cells with more than three nuclei are considered osteoclasts. Five fields of view were randomly selected, and the percentage of osteoclast area was calculated using Image J software (osteoclast area/field area × 100%).

**4.5 Flow Cytometry Analysis**

Cell grouping, interventions, and culture conditions were the same as before. After different treatments, RAW264.7 cells were resuspended in BD Pharmingen staining buffer (cat# 554657; BD Biosciences). Cells were incubated with FC block (cat# 553141; BD Biosciences) for 20 minutes. Subsequently, cells were washed and resuspended in 100 μL of BD Pharmingen staining buffer and incubated with APC Rat Anti-Mouse CD86 (cat# 560582; BD Biosciences), PE 647 Rat Anti-Mouse CD206 (cat# 565250; BD Biosciences) on ice for 30minutes. Cells were washed three times with staining buffer and resuspended in staining buffer. Cells were centrifuged and resuspended in staining buffer for FACS analysis (FACS Celesta; BD Bioscience, San Jose, USA). FACS data analysis was performed using FLOWJOTM Software.

**4.6 Alkaline Phosphatase Staining**

Cell grouping and culture conditions were the same as before. Osteogenic induction of PDLSCs Conditioned medium (CM) of the cocultured cells was harvested and mixed (1:1) with a-MEM culture medium (containing 10% FBS and 1% penicillin/streptomycin) supplemented with the osteogenic ingredients (dexamethasone (0.1 mM), ascorbic acid (50 mg/mL) and b-glycerophosphate (10 mM)). The above medium was used to incubate PDLSCs and changed every 2 days. After 7 days, quantitative real-time polymerase chain reaction (RT-qPCR) and western blotting were performed ALP staining (Beyotime Biotechnology) was performed on day 7 of the cell culture. The PDLSCs were fixed with 4% paraformaldehyde for 15 min, incubated for 45 min at 37 °C with the addition of staining solution, and excess dye was washed with distilled water. After drying, the staining results were observed under an inverted light microscope and photographed.

**4.7 Alizarin Red Staining**

Cell grouping, interventions, and culture conditions were the same as chapter 4.6. After day 21 of osteogenesis induction, ARS was used to observe the formation of mineralized nodules. Briefly, the PDLSCs were fixed in 4% paraformaldehyde for 30 min and washed thrice with PBS. ARS solution (Beyotime Biotechnology) was added and incubated at 37 °C for 45 min. The excess staining solution was then removed with distilled water. After drying, the cells were observed under an inverted light microscope and photographed.

**5. *In Vivo* Therapeutic Effects of Emb@CMCS-OD**

Animal experiments were conducted in accordance with the approved protocol by Institutional Animal Care and Use Committee (IACUC) of Sun Yat-Sen University (SYSU-IACUC-2022-002367) and complied with the NIH Guide for the Care and Use of Laboratory Animals (NIH Publication No. 8023, revised 1978).

**5.1 Establishment of Rat Periodontitis Model and Local Medication Injection**

30 SD rats (6-8 weeks, weighing 150 ± 20g) were randomly divided into four groups (*n* = 6), including healthy rats (Health), untreated periodontitis rats (Control), periodontitis rats treated with CMCS-OD only (Hydrogel), periodontitis rats treated with Embelin only (Emb), and periodontitis rats treated with Emb@CMCS-OD (Emb@Hydrogel). At first day, to induce experimental periodontitis, a 3-0 silk ligature was chosen to place around the maxillary second molar after rats were anesthetized by pentobarbital sodium (40 mg/kg body weight). At day 9^th^, microinjections were performed in periodontal tissues using an microneedle after anesthetizing. The Embelin group was injected with a 250 μM drug solution (200 μL). For the Emb@Hydrogel and Hydrogel groups, pre-hydrogel solution was mixed using a special premix adapter, and 200 μL was injected into specified positions in periodontal tissues totally (as mentioned above). The untreated periodontitis group was injected with an equal volume of PBS solution at the same positions.

**5.2 Gingival Index (GI) Measurement**

Intraperitoneal injection of pentobarbital sodium (40 mg kg^-1^ body weight) was administered. Then, using a probe, the gingival condition of the 6 sides of the maxillary second molar in each group was meticulously examined 4 weeks post-recovery. The GI was scored as follows: 0 points indicated normal gingiva with no signs of inflammation; 1 point indicated mild inflammation, and mild swelling without bleeding upon probing; 2 points indicated moderate inflammation, with bleeding upon probing; 3 points indicated severe inflammation, and a tendency for spontaneous bleeding.

**5.3 The pH value of Gingival crevicular fluid (GCF) in rats**

(1)Preparation: pH indicator paper (LH310, Luheng Biology) was used to record the pH values of the molar periodontal pockets of the rats, which can measure pH values ranging from 4.5 to 9.0. (2)Sampling: A Hamilton microinjector (700 series, Yuyan Biology) was used to carefully extract GCF samples from the periodontal pockets of rat. Before extraction, the area was rinsed with physiological saline to remove potential contaminants, and then dried with sterile cotton swabs to ensure that the sample was not contaminated with saliva. (3)Testing: The extracted gingival crevicular fluid samples were evenly moistened with pH test paper. After about 5 seconds, the color change was observed. According to the color chart provided with the test paper, the color was matched with the corresponding pH value. (4)Recording: The pH value of each sample was recorded and the data was statistically analyzed and presented in a bar graph.

**5.4 Micro-CT Scanning**

Micro-CT (Siemens Inveon PET/CT) was used for *in vivo* analysis of the rat periodontal tissue samples from periodontitis model (80 kV, 500 μA). Three-dimensional (3D) views were reconstructed using Inveon Research Workplace following the same standards, with all cusps at the same level and the occlusal plane not visible from the buccal or palatal side. The vertical distance between the cementoenamel junction (CEJ) and the alveolar bone crest (AB), representing the degree of alveolar bone loss, was measured. Data were summarized and presented as bar graphs, 2D sectional images, and 3D reconstructions.

**5.5 Hematoxylin and Eosin (H&E)**

The fixed rat periodontal tissue samples were decalcified in 10% ethylenediaminetetraacetic acid (EDTA) for two months. The samples were then dehydrated in a series of graded ethanol solutions. After being cleared in xylene, the specimens were embedded in paraffin to obtain 5 μm thick sections. H&E staining was performed to evaluate periodontal regeneration. The sections were rehydrated with distilled water. After rehydration, the sections were stained with hematoxylin for 1 minute, quickly rinsed to remove excess stain, then differentiated in 1% hydrochloric acid-ethanol solution for 2-5 seconds to remove non-nuclear hematoxylin staining, followed by rinsing under running tap water for 15 minutes for bluing. The sections were then stained with eosin for 10 seconds and rinsed with distilled water until the desired intensity of staining was achieved. The sections were dried in a 37°C oven, cleared in xylene twice, each for 5 minutes, and mounted with a neutral balsam cover slip. The slides were air-dried in a fume hood and images were captured using a slide scanner.

**5.6 Tartrate Resistant Acid Phosphatase (TRAP) Staining of Paraffin Tissue Sections**

The samples process as same as above. TRAP staining was performed to evaluate osteoclast formation. The final sections were rehydrated with distilled water. After rehydration, TRAP staining solution was freshly prepared according to the kit instructions and applied to the tissue surface. The sections were incubated in a 37°C oven for 30 minutes.

Under a light microscope, TRAP-positive cells appeared wine-red, and cells with more than three nuclei were considered osteoclasts. At high magnification, five random fields of view were selected, and the percentage of osteoclast area was calculated using ImageJ software (National Institutes of Health, Bethesda, Maryland, USA) (osteoclast area/field area × 100%).

**5.7 Immunohistochemical Staining**

The samples process as same as above. Following rehydration, sections were immersed in 95°C pre-heated sodium citrate antigen retrieval solution and placed in a hot air oven for antigen retrieval at 95°C for 30 minutes, then allowed to cool to room temperature. After antigen retrieval, tissue sections were circled with an immunohistochemistry pen. The endogenous peroxidase blocking, serum blocking, biotin blocking, and streptavidin blocking from the HRP-DAB IHC rabbit detection kit were sequentially applied to the sections as per the kit's manual.

Following this, sections were incubated overnight at 4°C in a humid chamber with Anti-IL-1beta(ab283818, Abcam, 1:100), Osteocalcin(MA1-20786, Thermofisher, 1:200) and Anti-TNF alpha(ab307164, Abcam, 1:100). The next day, sections were washed three times with PBS for 5 minutes each, followed by sequential application of biotin-labeled secondary antibodies and streptavidin-biotin-horseradish peroxidase (HRP) complex as per the kit's instructions. Color development was conducted using freshly prepared DAB chromogen provided in the kit, with color development time controlled under the microscope. Pure water was used to wash and terminate the color development. After coloring, hematoxylin was used for nuclear counterstaining, ensuring the staining was not overly intense. For image acquisition, five random fields were selected and analyzed to create quantitative bar graphs.

**5.8 Enzyme-linked Immunosorbent Assay (ELISA) for Tissue Inflammatory Factor Expression**

Collected rat periodontal tissue samples were thoroughly cleaned to remove blood, fat, and other impurities. RIPA buffer was added, and tissues were homogenized using a tissue homogenizer or homogenizer at 4°C. The supernatant containing proteins was collected after centrifugation, avoiding disturbing the sediment. Supernatants from each group were measured for Tumor Necrosis Factor α (TNF-*α*) and Interleukin 1β (IL-1*β*) using ELISA kits (Thermo Fisher Scientific) as per the instructions of kit. Absorbance for each well was measured using a spectrophotometer, with five replicates per group. Concentrations of inflammatory factors in the samples were calculated based on the standard curve.

All reagents and solvents were of biochemical reagent (BR) grade or analytical reagent (AR) grade and were used as received unless otherwise specified.

**6. Statistical Analysis**

All quantitative data were performed as the mean ± SD. In terms of evaluating outliers, we discarded any data values that deviated more than two standard deviations from the mean. The statistical analysis was calculated by one-way analysis of variance (ANOVA) followed by Turkey’s multiple comparison tests or Student’s t-test via GraphPad Prism 8.0 software. The sample size (n) for each statistical analysis has been mentioned in the corresponding “figure legends.” The level of significance was pre-set alpha level of 0.05. Values of *p* < 0.05 were considered statistically significant (* *p* < 0.05; ** *p* < 0.01; *** *p* < 0.001). Each experiment was repeated a minimum of three times.

**Reference**

[1] V. G. Muir, J. A. Burdick, *Chemical reviews* **2021**, *121*, 10908.

[2] Y. Jia, X. Yan, J. Li, *Angewandte Chemie (International ed. in English)* **2022**, *61*, e202207752.

[3] Y. Wang, Q. Lv, Y. Chen, L. Xu, M. Feng, Z. Xiong, J. Li, J. Ren, J. Liu, B. Liu, *Acta pharmaceutica Sinica. B* **2023**, *13*, 284.

[4] Y. Wang, J. Lee, J. R. Werber, M. Elimelech, *Science advances* **2020**, *6*, eaax5253.


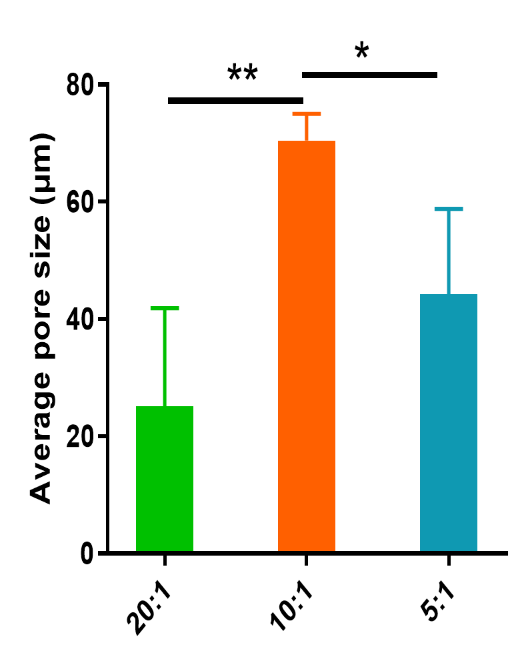


**Figure S1.** Pore size distribution of CMCS-OD with different mixing ratios. Data were expressed as mean ± SD. *n =* 3, * *p* < 0.05; ** *p* < 0.01.


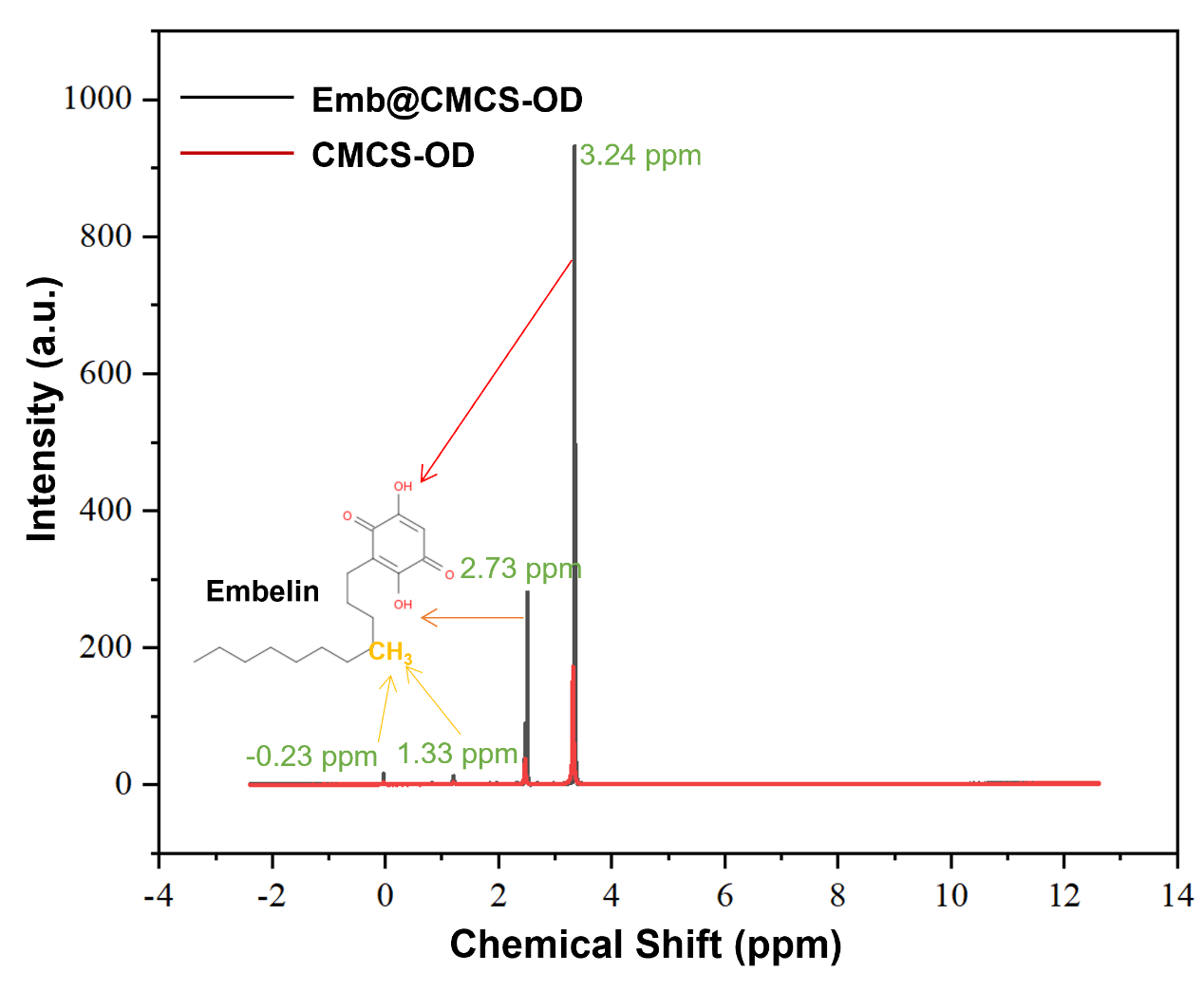


**Figure S2.** Nuclear Magnetic Resonance (NMR) characterization of CMCS-OD and Emb@CMCS-OD.


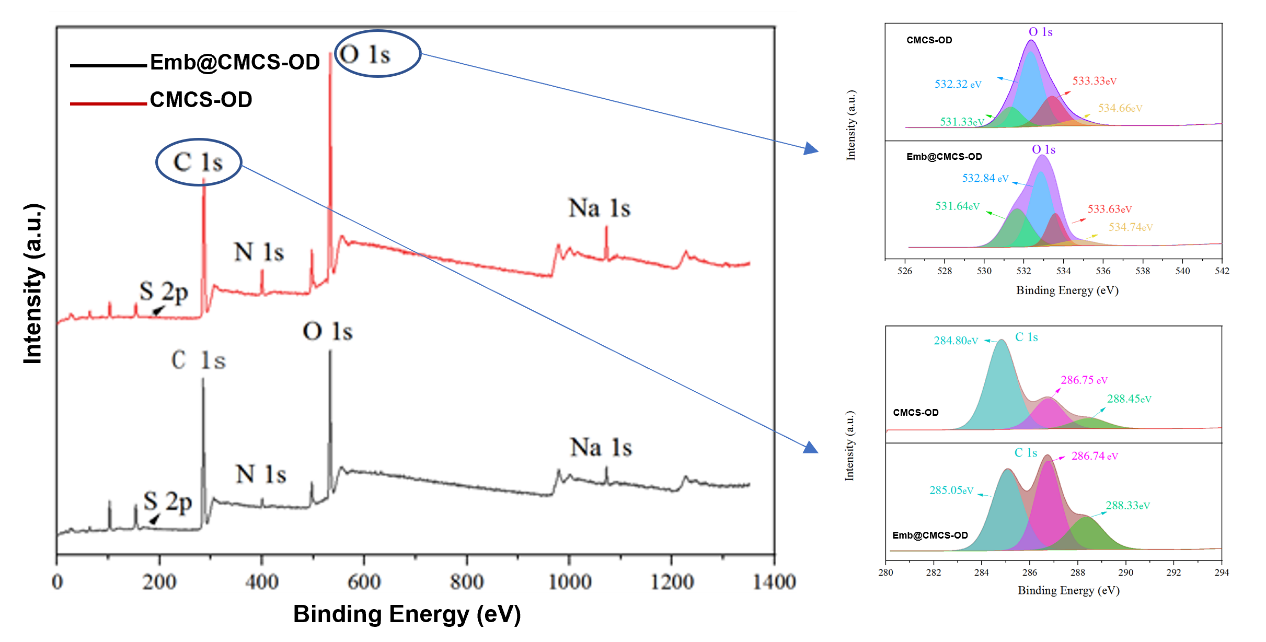


**Figure S3.** X-ray photoelectron spectroscopy (XPS) characterization of CMCS-OD and Emb@CMCS-OD.

**
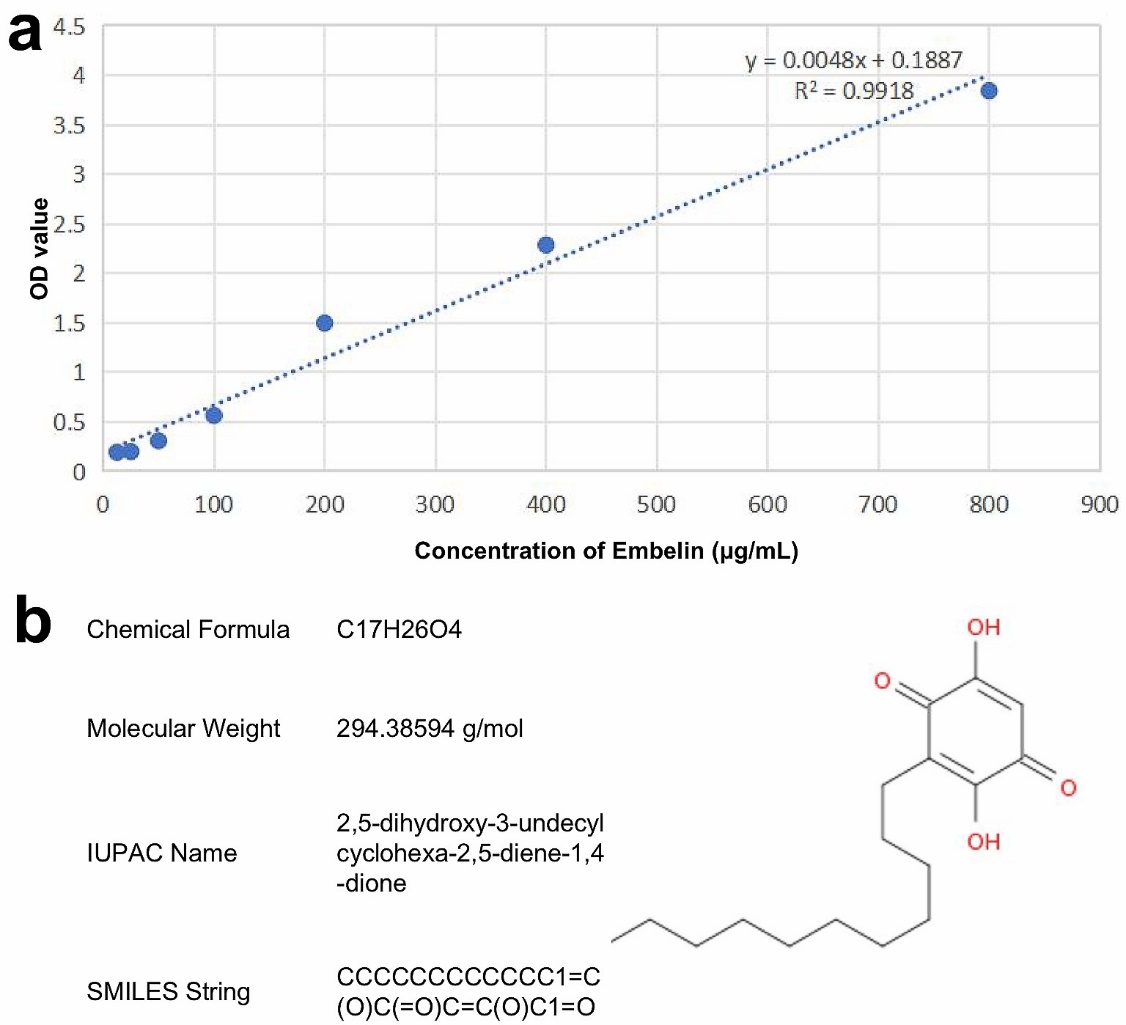
**

**Figure S4.** (a). Standard curve of Embelin. (b). The molecular structure of Embelin.
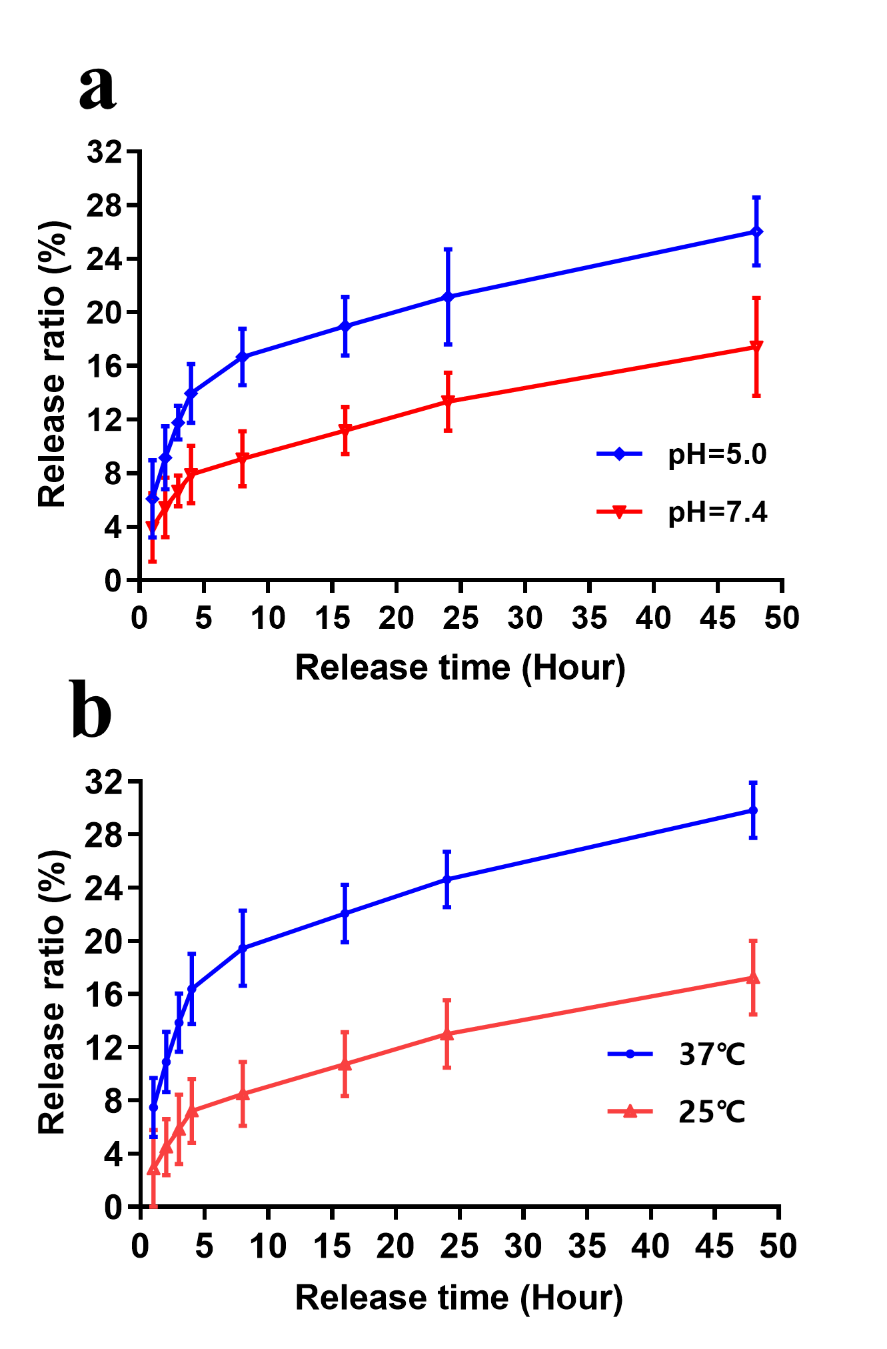


**Figure S5.** (a). The release kinetics of Embelin from Emb@CMCS-OD over 48 h under different pH conditions (5.0 and 7.4). (b). The release kinetics of Embelin from Emb@CMCS-OD over 48 h under different temperatures (25°C and 37 °C). Data were expressed as mean ± SD. *n =* 3.


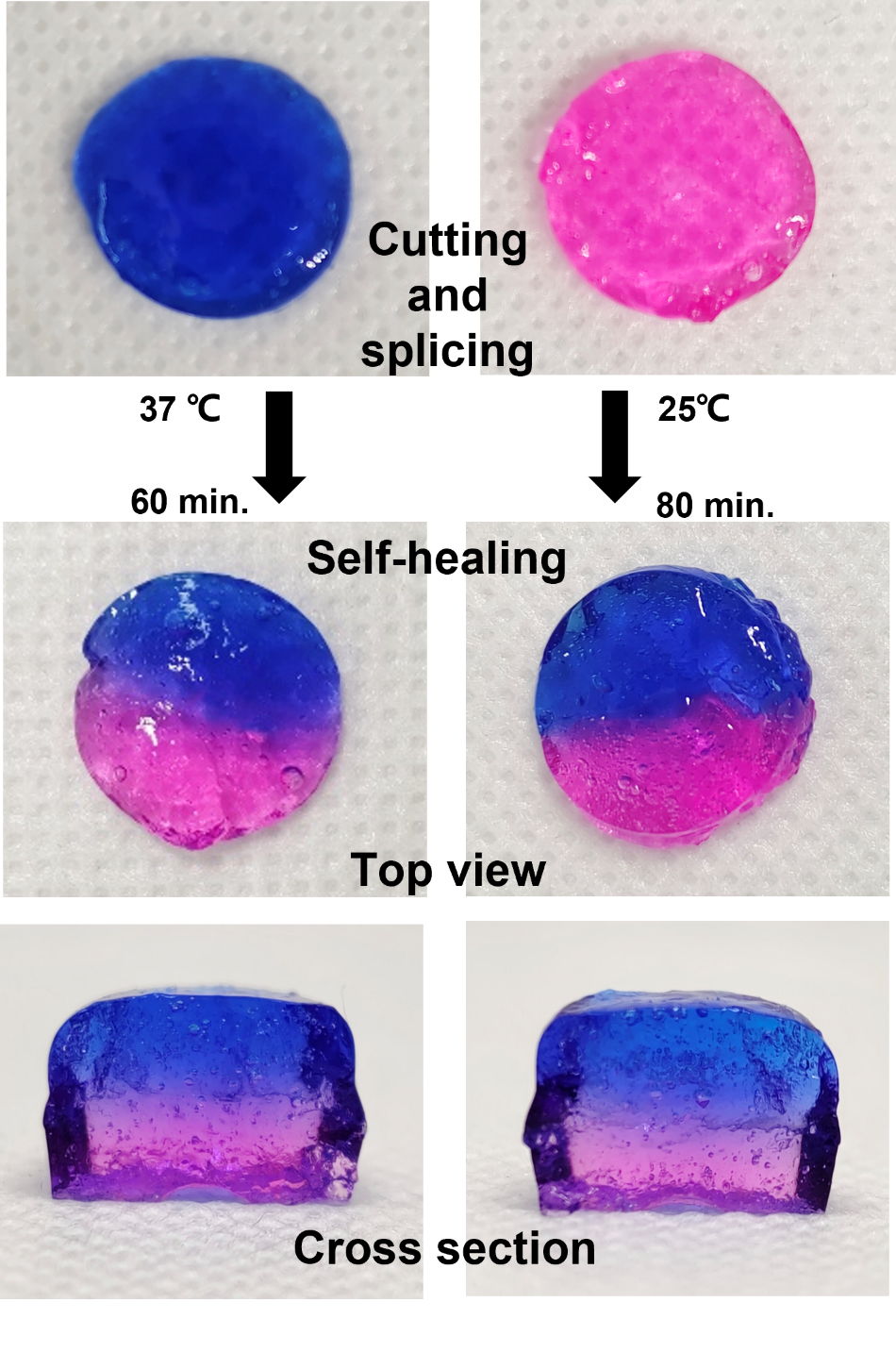


**Figure S6.** Photographs demonstrated the self-healing behavior of the CMCS-OD under different temperatures (37°C and 25 °C).


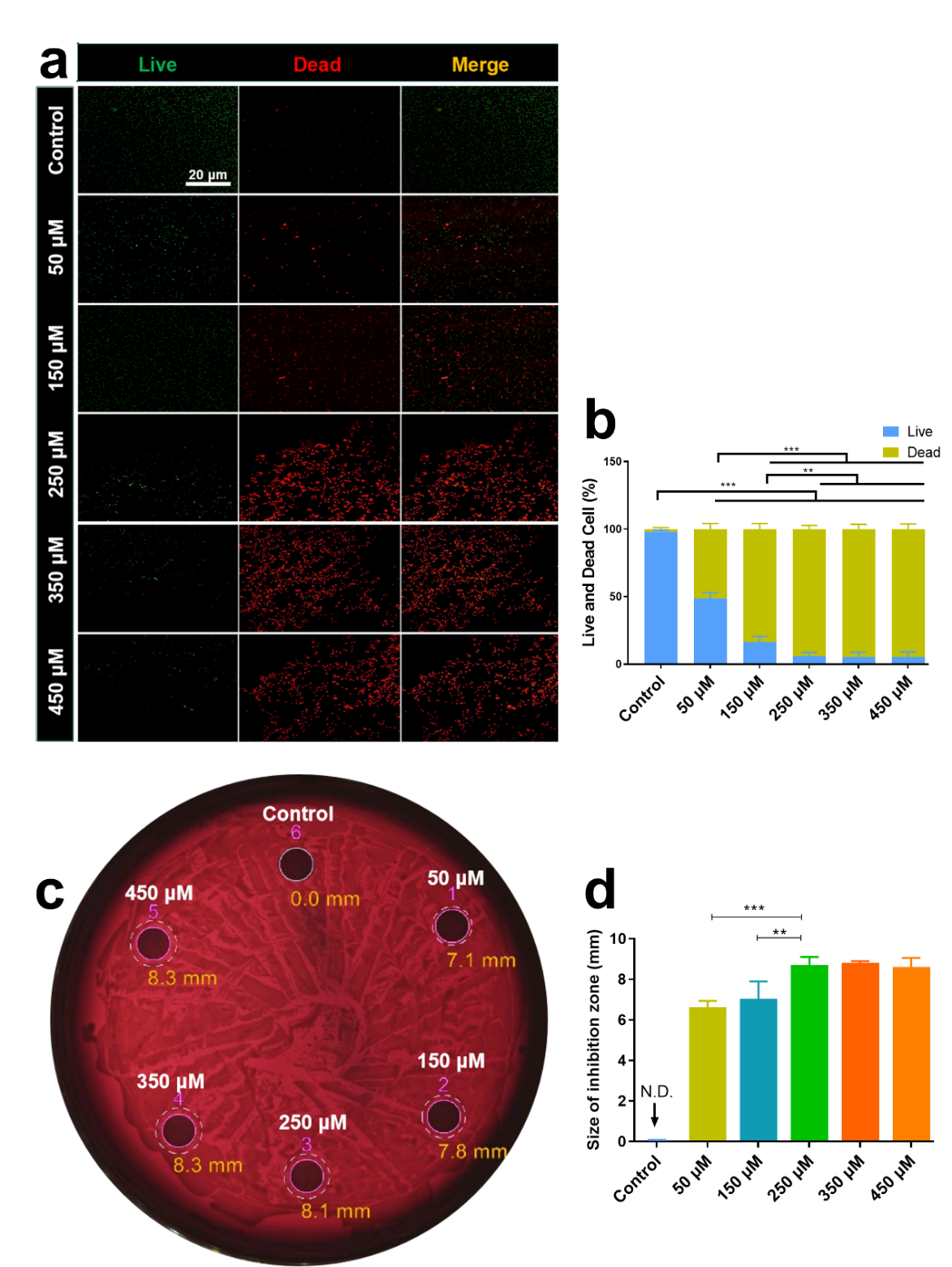


**Figure S7.** Antibacterial Effect of Embelin on *P. gingivalis*. (a, b). The antibacterial effect of Embelin was evaluated by live-dead bacteria staining and quantitative analysis of representative microphotographs. (c, d). The antibacterial effect of Embelin was evaluated by disc diffusion susceptibility test, and quantitative analysis of the size of inhibition zone. Data were expressed as mean ± SD. *n =* 3, * *p* < 0.05; ** *p* < 0.01; *** *p* < 0.001; ND indicates not detected.


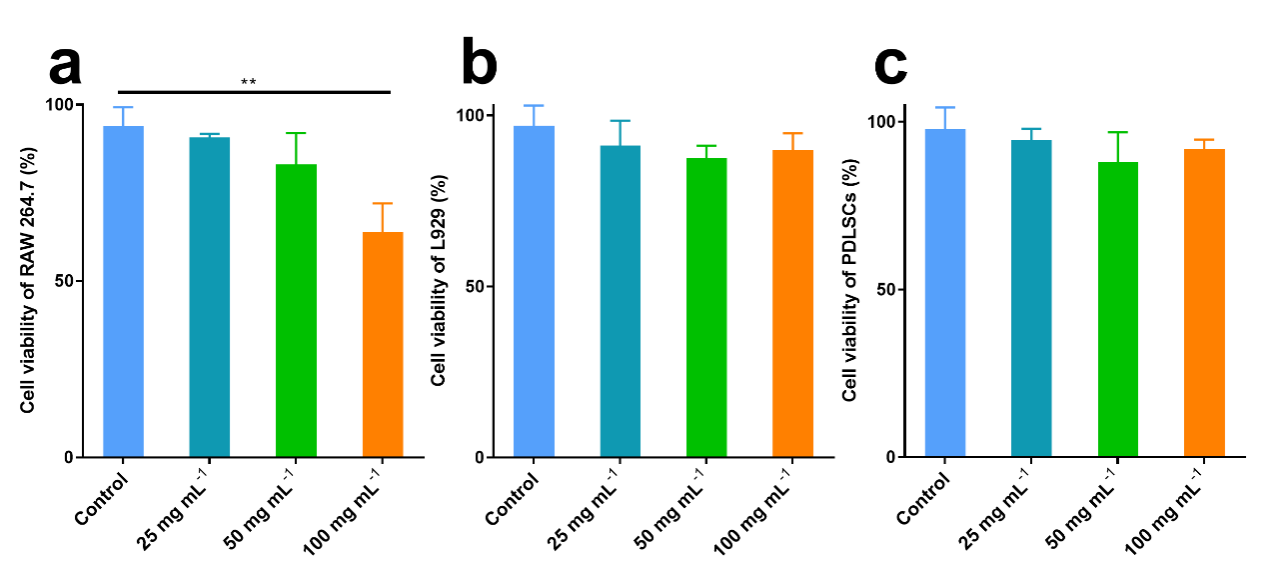


**Figure S8.** Biocompatibility of CMCS-OD. (a). Viability of RAW264.7 cells was evaluated by the CCK-8 assay. (b). Viability of L929 cells was evaluated by the CCK-8 assay. (c). Viability of PDLSCs was evaluated by the CCK-8 assay. Data were expressed as mean ± SD. *n =* 3, * *p* < 0.05; ** *p* < 0.01.


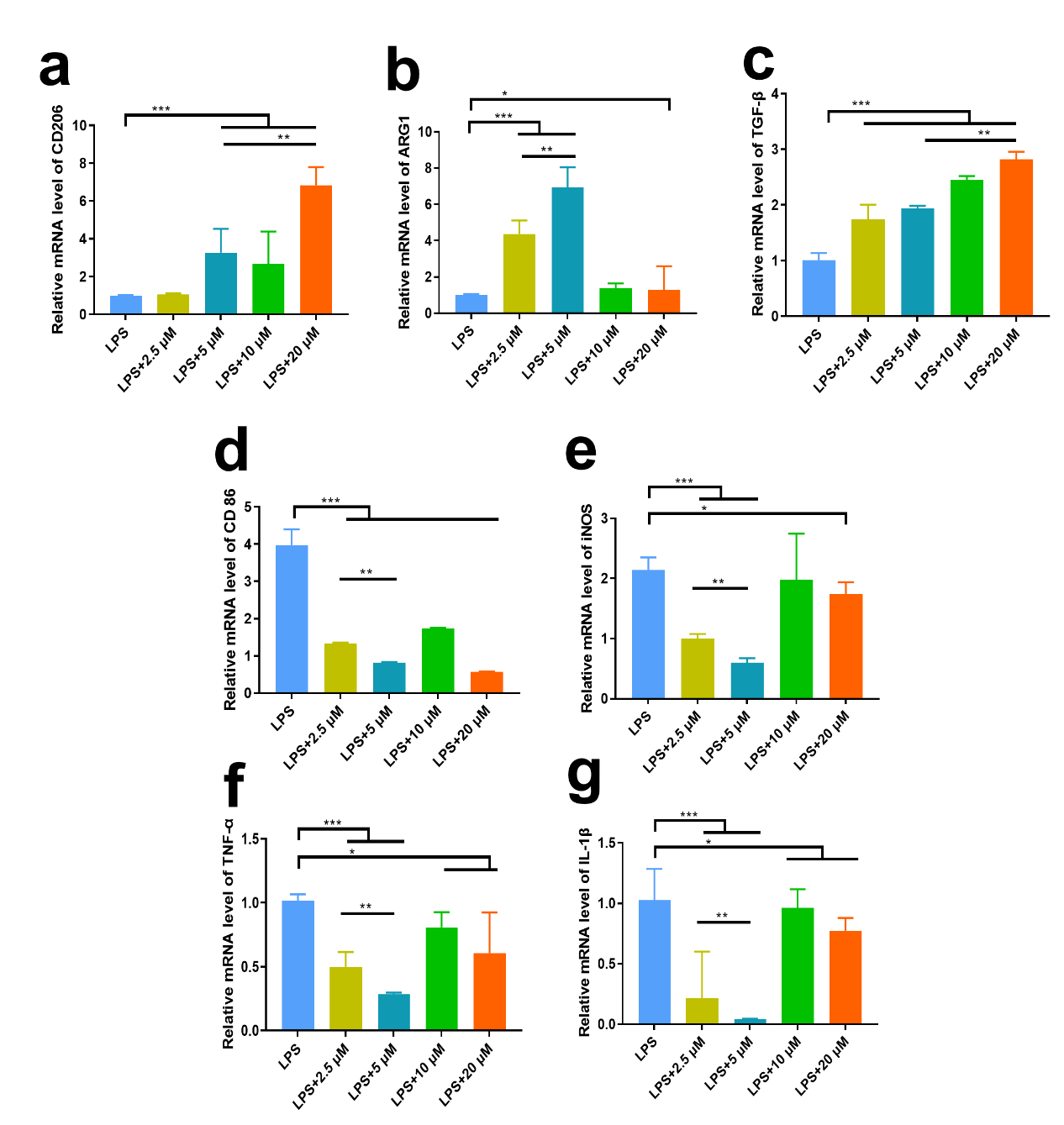


**Figure S9.** Optimal concentration of embelin with anti-inflammatory activity. (a, b, c). The mRNA expression of M2 macrophage–phenotype markers (*CD206, ARG1,TGF-β*). (d, e). The mRNA expression of M1 macrophage–phenotype markers (*CD86,iNOS*). (f, g). The mRNA expression of pro-inflammatory cytokines (*TNF-α and IL-1β*). Data were expressed as mean ± SD. *n =* 3, * *p* < 0.05; ** *p* < 0.01; *** *p* < 0.001.


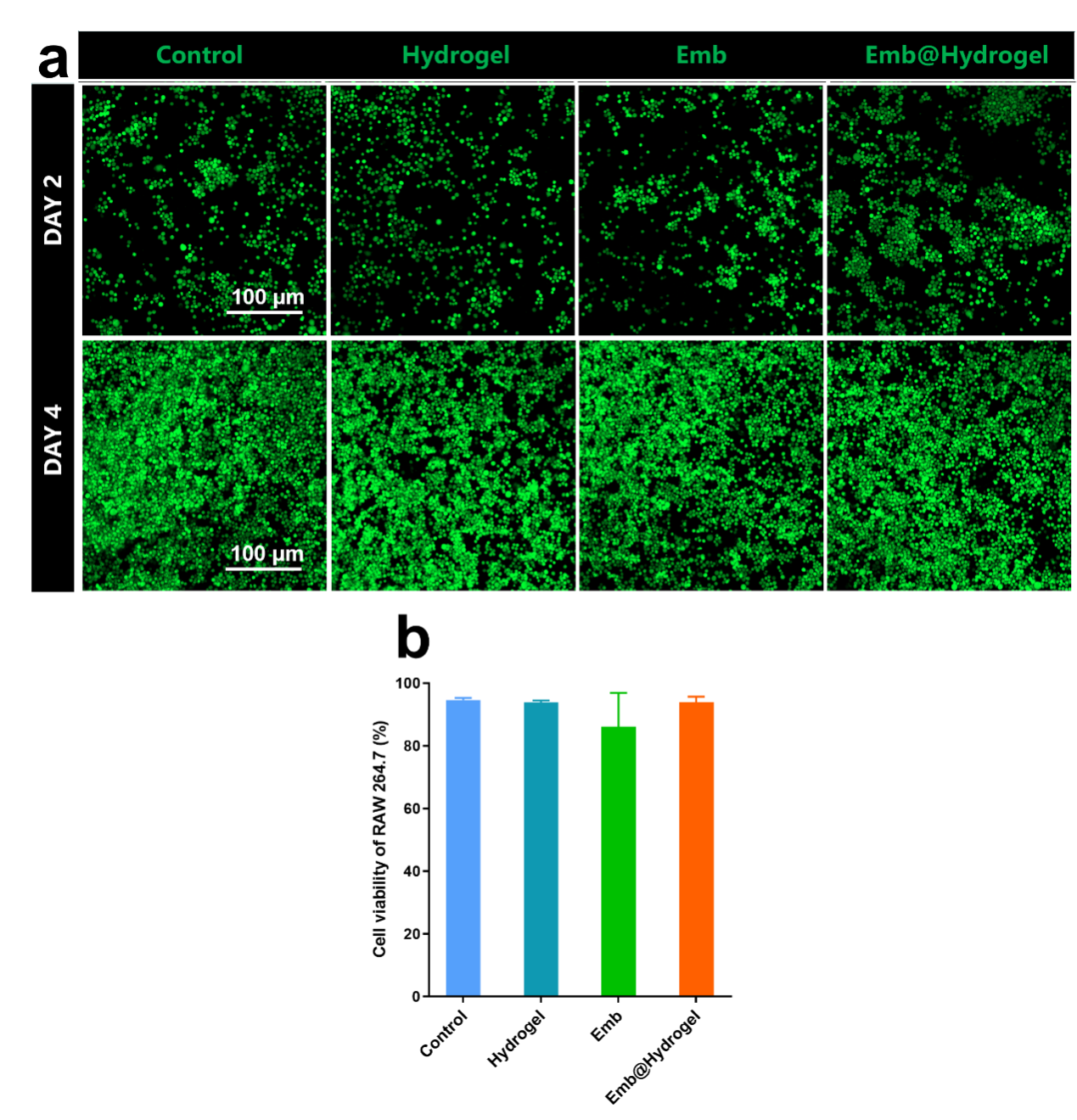


**Figure S10.** Biocompatibility of Emb@CMCS-OD. (a). Cell proliferation of RAW264.7 was evaluated by LIVE/DEAD (green/red) staining. RAW264.7 without any treatment served as control. (b). Viability of RAW264.7 was evaluated by the CCK-8 assay. Data were expressed as mean ± SD. *n =* 3.


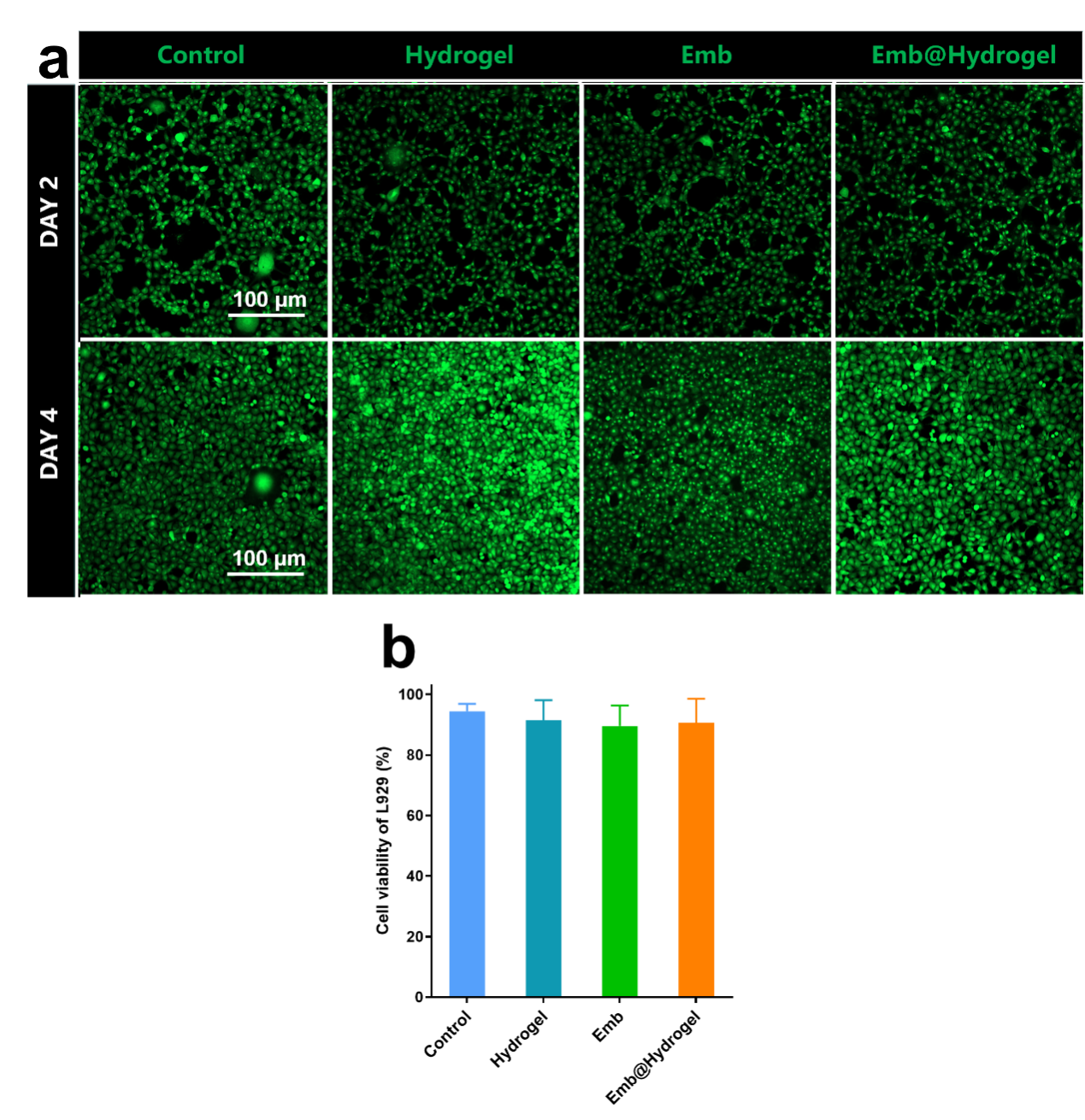


**Figure S11.** Biocompatibility of Emb@CMCS-OD. (a). Cell proliferation of L929 was evaluated by LIVE/DEAD (green/red) staining. L929 without any treatment served as control. (b). Viability of L929 was evaluated by the CCK-8 assay. Data were expressed as mean ± SD. *n =* 3.


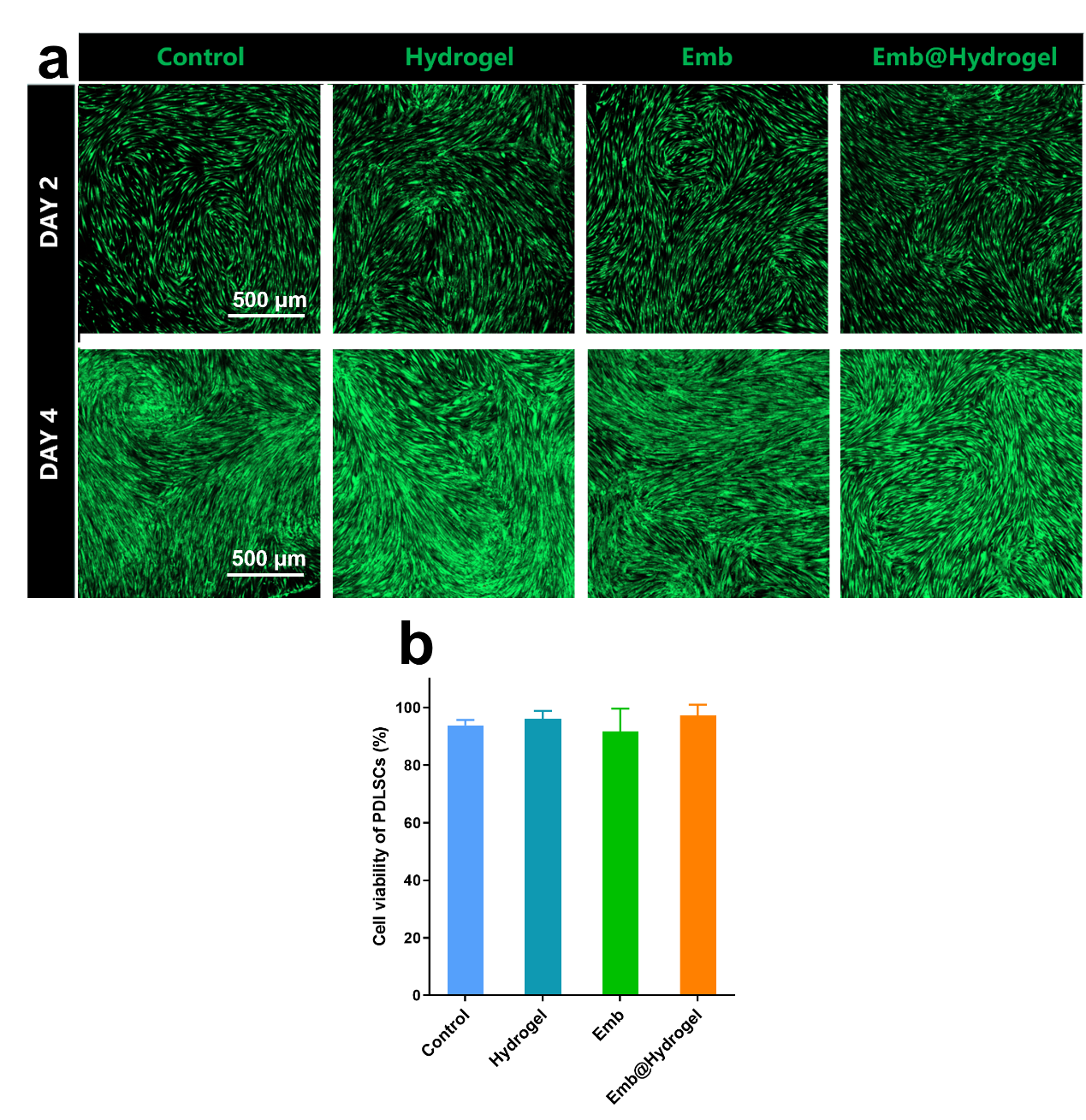


**Figure S12.** Biocompatibility of Emb@CMCS-OD. (a). Cell proliferation of PDLSCs was evaluated by LIVE/DEAD (green/red) staining. PDLSCs without any treatment served as control. (b). Viability of PDLSCs was evaluated by the CCK-8 assay. Data were expressed as mean ± SD. *n =* 3.


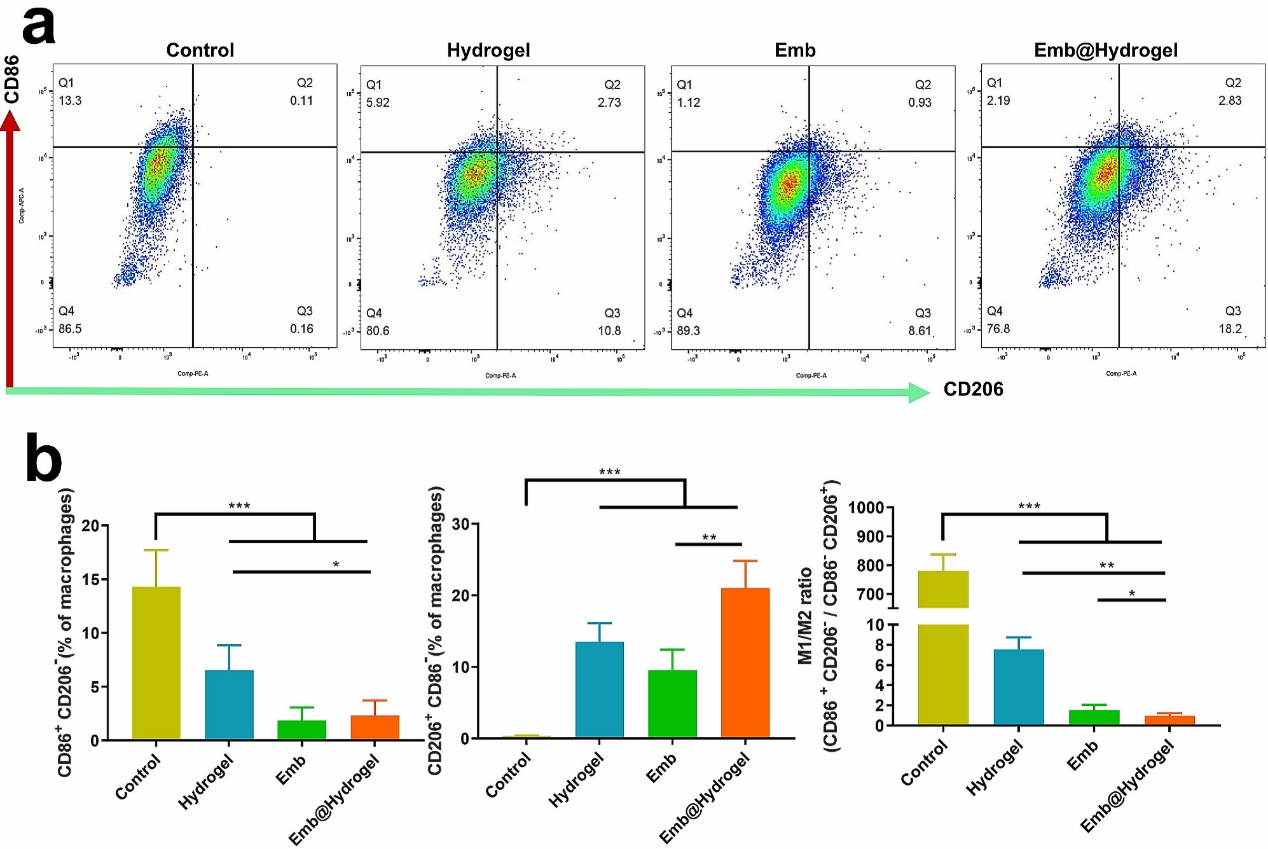


**Figure S13**. The effect of Emb@CMCS-OD on phenotypic regulation on macrophage. (a). Flow cytometry analysis of RAW264.7 cells including the level of CD86 and CD206. (b). Quantitative analysis of the CD86/CD206 ratios. Data was expressed as mean ± SD. *n* = 3, * *p* < 0.05; ** *p* < 0.01; *** *p* < 0.001.


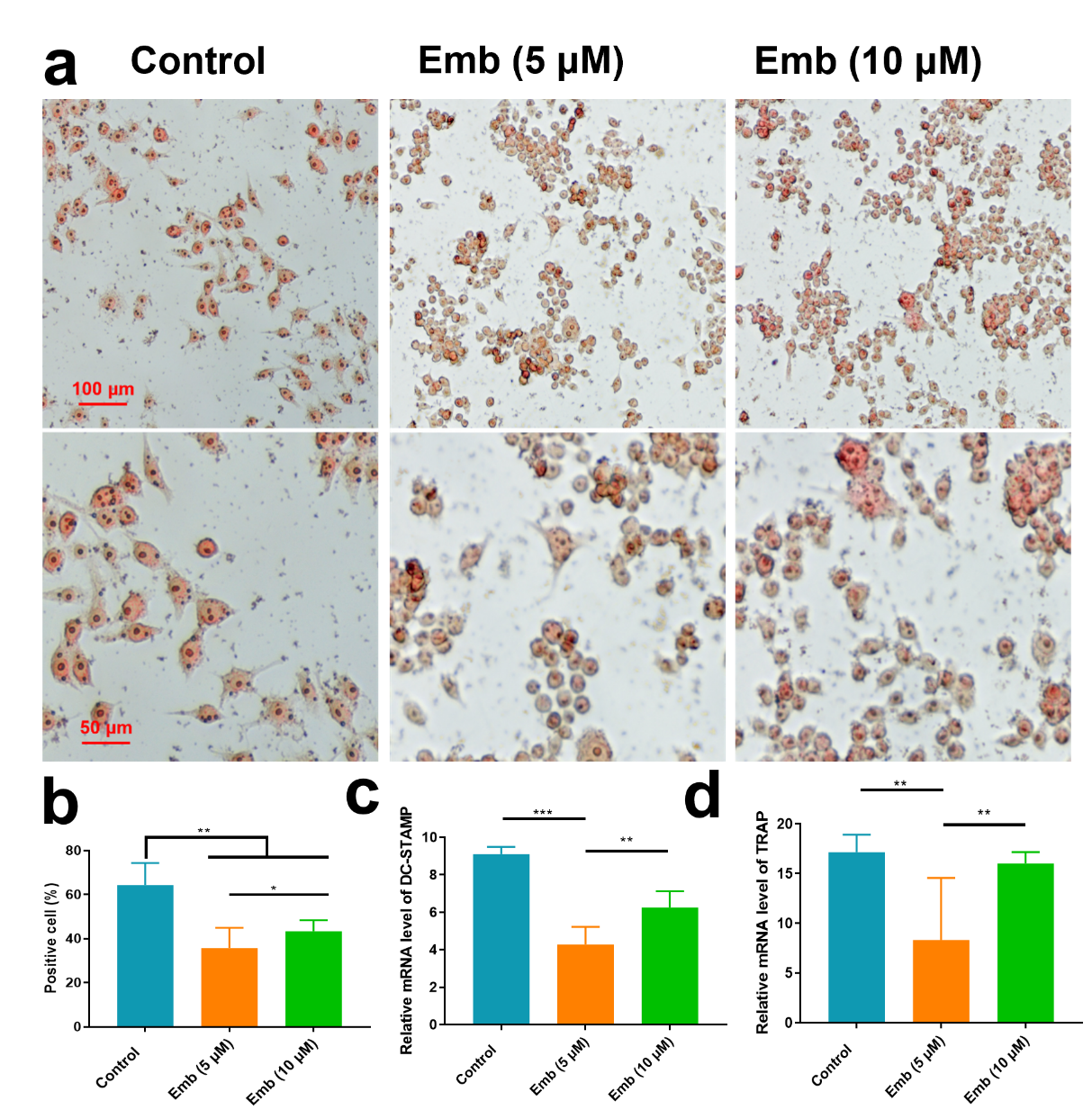


**Figure S14.** The inhibitory effect of Embelin on the differentiation of RAW264.7 into osteoclasts (a, b). Representative microphotographs of by tartrate-resistant acid phosphatase (TRAP) staining and quantitative analysis of the TRAP^+^ cells. (c, d). The mRNA expression (*DC-STAMP, TRAP*) of macrophages was evaluated by qRT-PCR. Data were expressed as mean ± SD. *n =* 3, * *p* < 0.05; ** *p* < 0.01; *** *p* < 0.001.


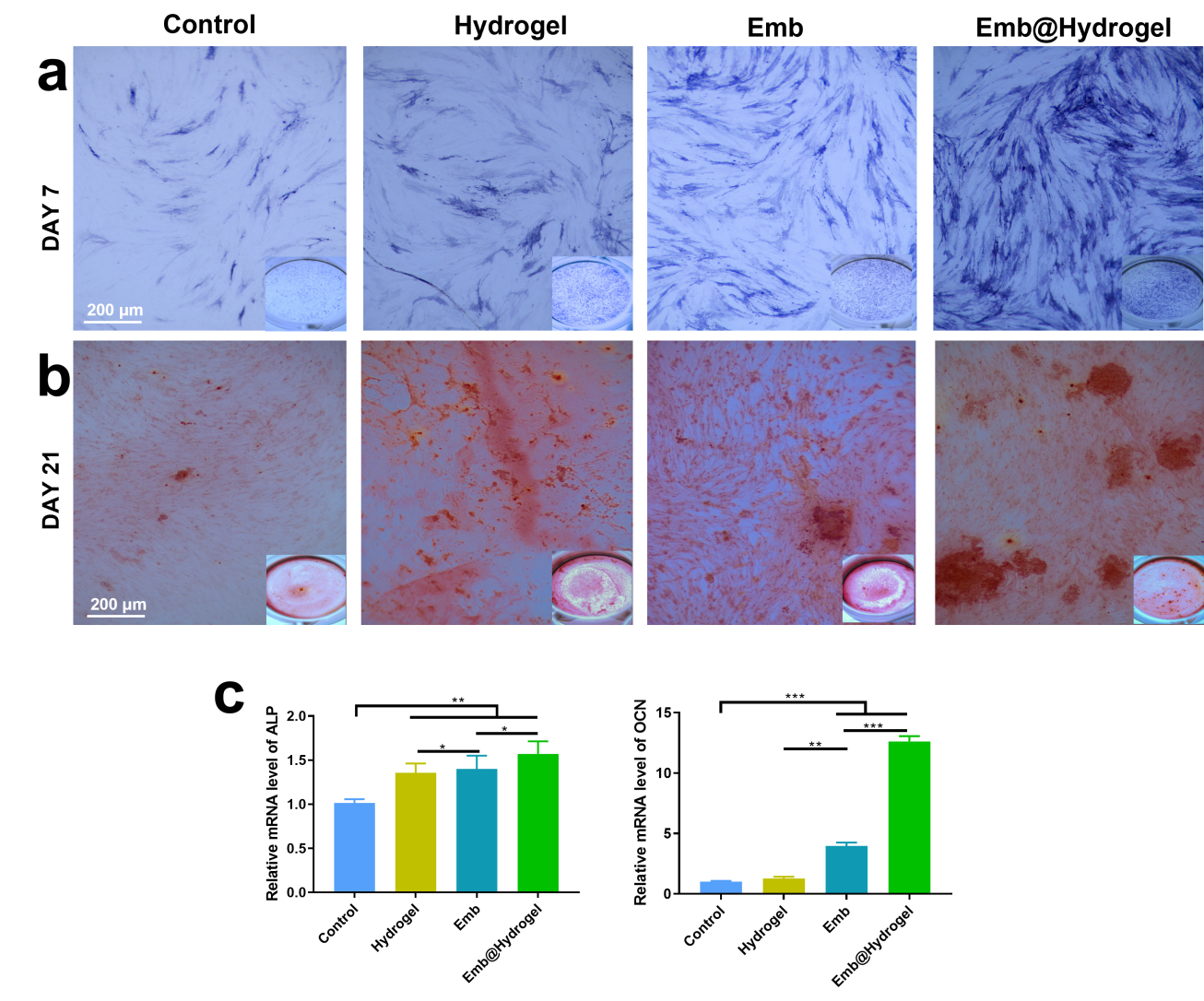


**Figure S15**. Emb@CMCS-OD accelerate the Osteogenic differentiation of PDLSCs under inflammatory conditions. (a). ALP staining of PDLSCs. (b). Alizarin red staining of PDLSCs. (c). The mRNA expression of osteogenesis markers (*ALP, OCN*) examined by qRT-PCR. Data was expressed as mean ± SD. *n* = 3, * *p <* 0.05; ** *p <* 0.01; *** *p <* 0.001.


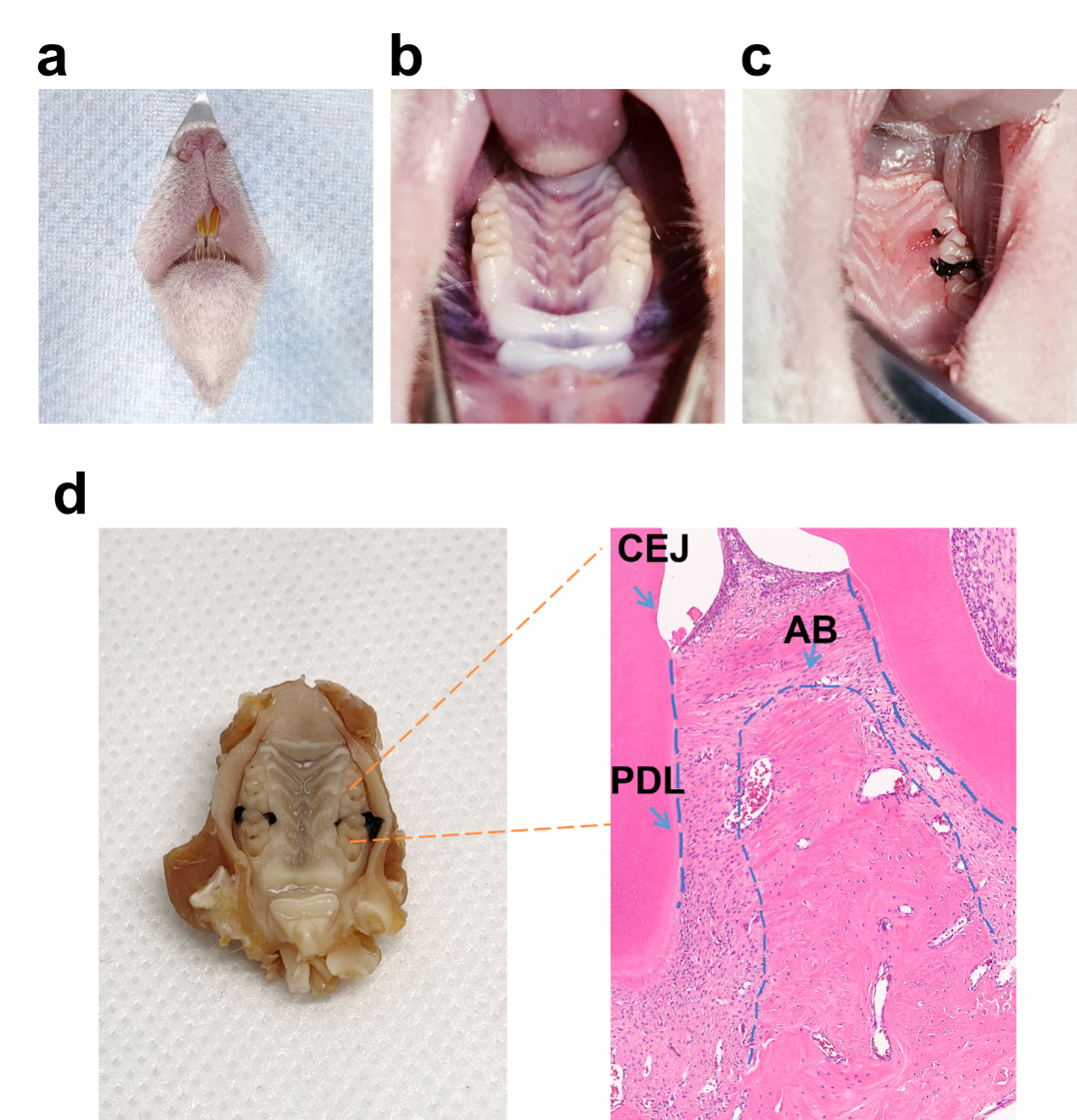


**Figure S16**. Scheme of ligation-induced periodontitis model. (a). SD rats were placed in the supine position after anesthesia. (b). Healthy periodontal tissue of rats. (c). On the 9th day after surgery, the 3-0 line was still staying the maxillary molars, and the gums were red and bleeding. (d). The main structures of periodontal tissue stained by H&E. (CEJ: Cemento-enamel junction. AB: Alveolar bone. PDL: Periodontal ligament.)


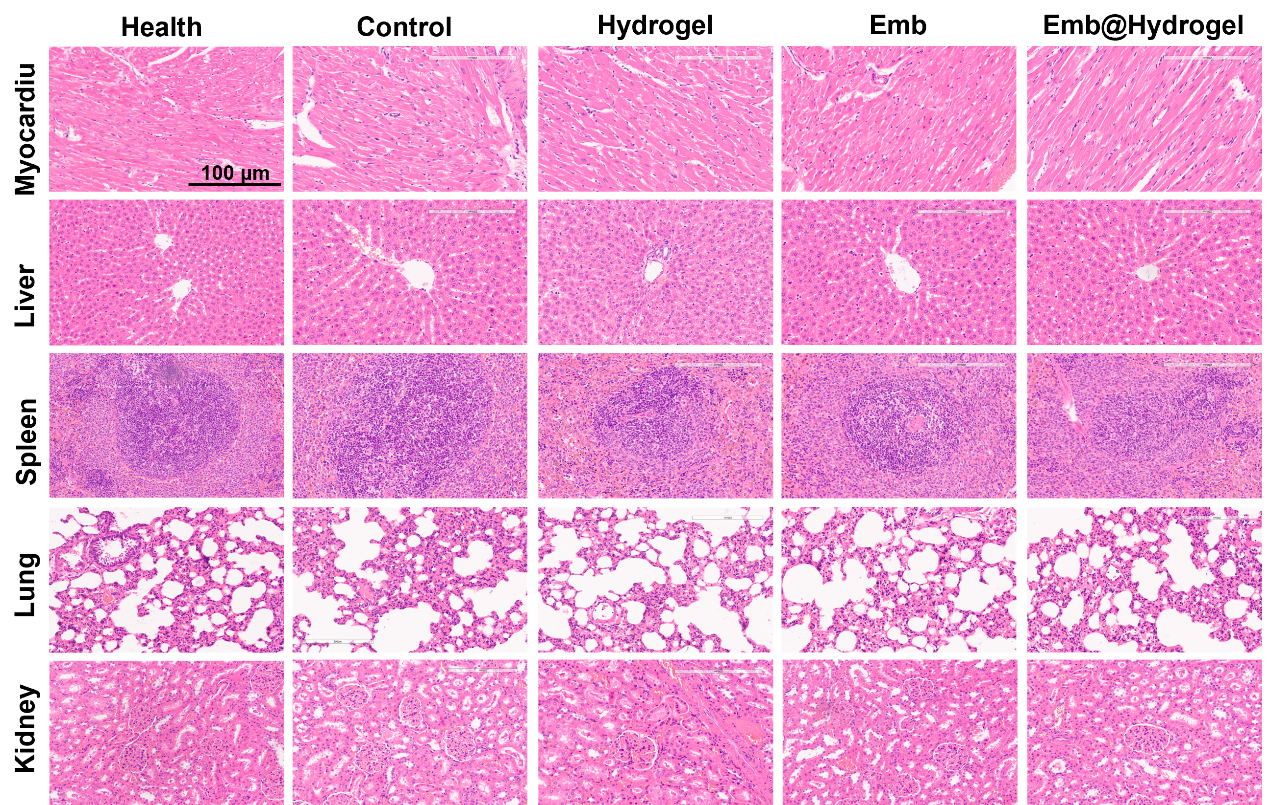


**Figure S17.** Representative microphotographs of H&E-stained slices of heart, liver, spleen, lung and kidney. *n* = 6.


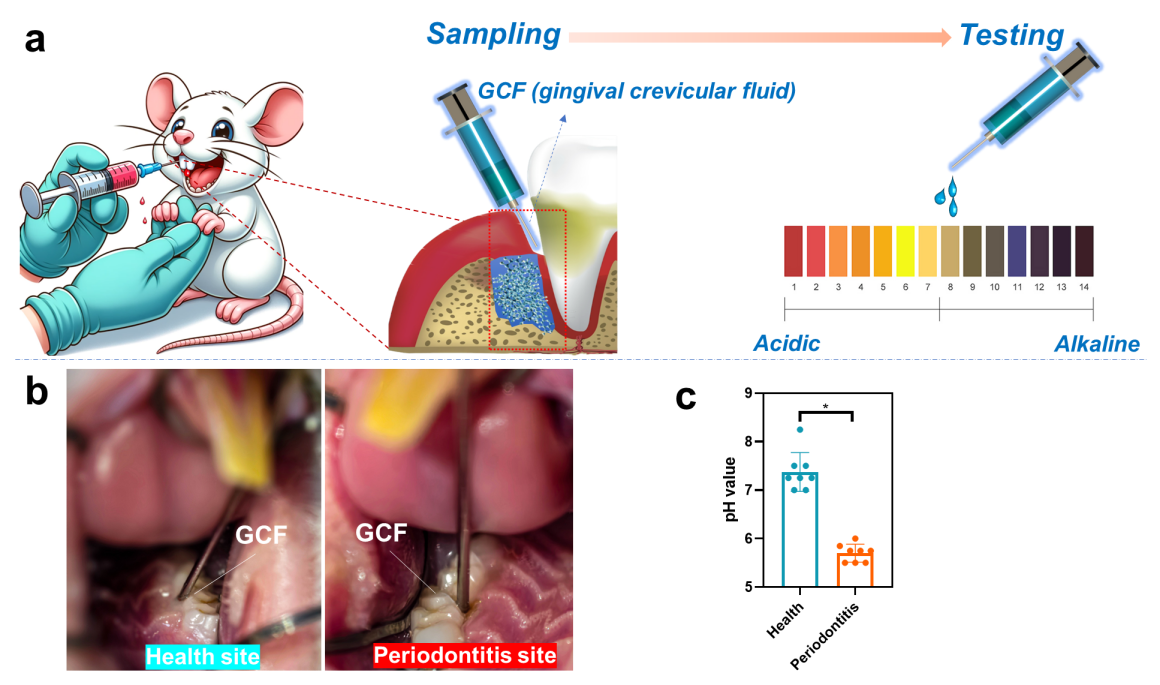


**Figure S18**. (a) Schematic diagram of gingival crevicular fluid samples collected from a rat periodontitis model and assayed for pH value. (b) Gingival crevicular fluid samples collected from healthy and periodontitis model rats. (c) Statistical bar graph of pH value data in healthy and periodontitis model rats. Data are expressed as mean ± SD. *n* = 8, * *p* < 0.05.


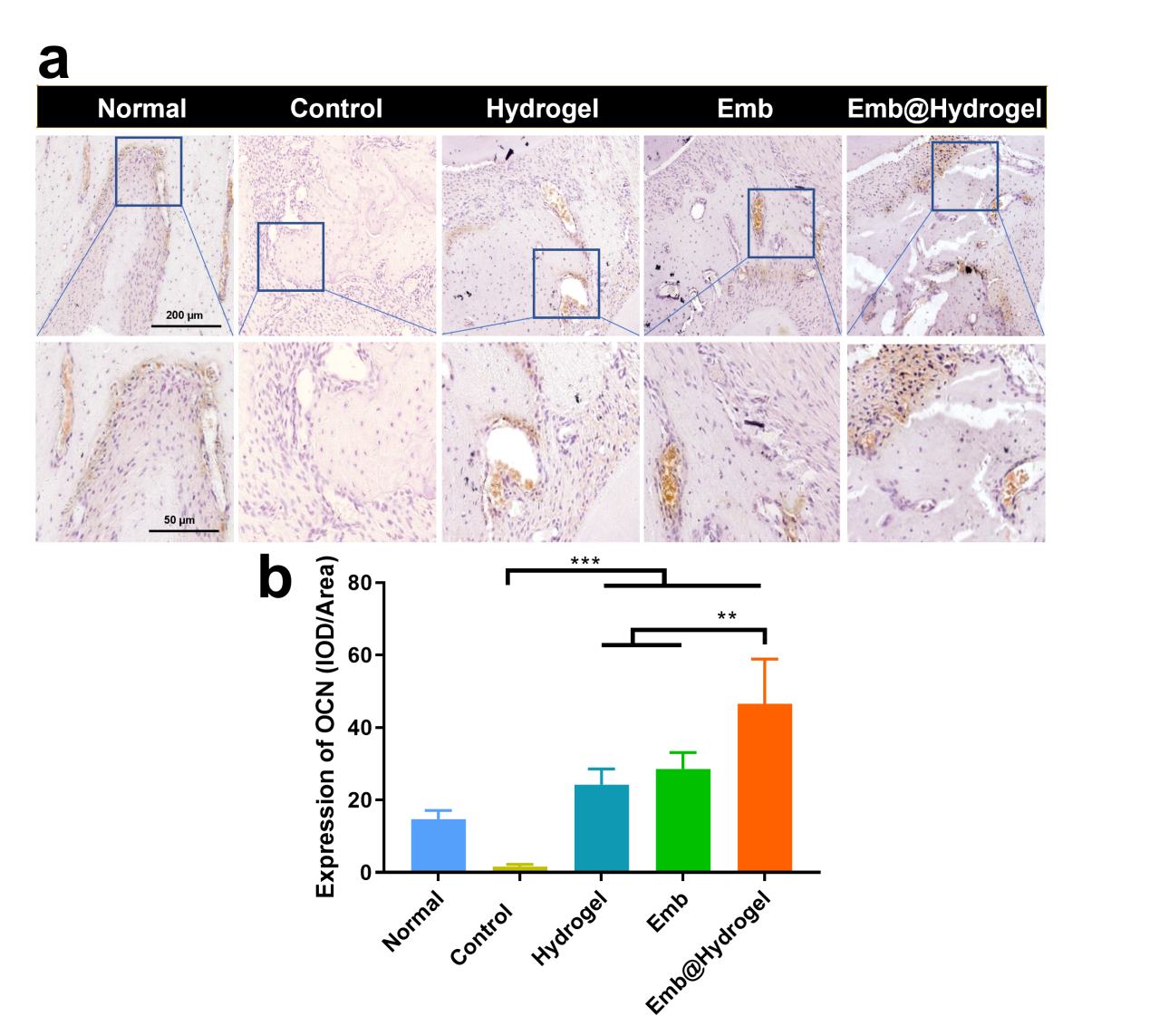


**Figure S19**. Enhanced osteogenesis induced by Emb@CMCS-OD *in vivo*. (a, b). The expression of OCN in periodontal tissue were examined by immunohistochemical staining and quantitative analysis of the representative photograph. Data was expressed as mean ± SD. *n* = 6, * *p <* 0.05; ** *p <* 0.01; *** *p <* 0.001.

**Table S1**. Summary of concentration of Embelin *in vitro* cell experiments.

| **Dosing Concentration** | **Optimal Concentration** | **Pathogen type** | **Effects** | **References** |
| --- | --- | --- | --- | --- |
| 1-3478 μM  (1-1024 μg mL^-1^) | 870 μM  (256 μg mL^-1^) | Drug-resistant *Pseudomonas* *aeruginosa* and *E.* *coli* | Significantly inhibited the growth of the two drug-resistant bacteria, the minimal inhibitory concentration can be reduced to 64 μg mL^-1^ when combined with ciprofloxacin | Khare, Tushar et al. *Environmental research* vol. 199 (**2021**): 111321. 1 |
| 5- 35 μM  (1.5-10.3 μg mL^-1)^ | 35 μM  (10.3 μg mL^-1^) | Bladder cancer cells | Inhibited cancer cell growth and inflammation | Fu, X et al. *Clinical & translational oncology : official publication of the Federation of Spanish Oncology Societies and of the National Cancer Institute of Mexico* vol. 18,3 (**2016**): 277-82. |
| 0-20 μM  (0-6 μg mL^-1^) | 15-20 μM  (4.5-6 μg mL^-1^) | Breast cancer cells | Inhibited cancer cell growth and reduced TNF-*α* expression by 4 fold | Dhanjal, Jaspreet Kaur et al. *BMC cancer* vol. 14 775. 22 Oct. **2014**, |
| 0.1-30 μM  (0.03-8.8 μg mL^-1^) | 10-30 μM  (3-8.8 μg mL^-1^) | Human keratinocytes | Reduced TNF-*α* expression by two-fold | Kalyan Kumar, G et al. *European journal of pharmacology* vol. 662,1-3 (**2011**): 63-9. |
| 0.1-10 μM  (0.03-3 μg mL^-1^) | 10 μM  (3 μg mL^-1^) | Leukocytes | Exerted anti-inflammatory effects by reducing PCG2 expression | Schaible, Anja M et al. *Biochemical pharmacology* vol. 86,4 (**2013**): 476-86. |
| 1-10 μM  (0.3-3 μg mL^-1^) | 5 μM  (1.5 μg mL^-1^) | Gastrointestinal epithelial cells | Protected IL-1*β* treated epithelial cells and reduced COX-2 expression, exhibiting anti-inflammatory effects | Lee, In-Seung et al. *Immunopharmacology and immunotoxicology* vol. 40,1 (**2018**): 83-90. |

**Table S2**. Primer sequence list.

| ***Gene*** | **Forward primer (5' → 3')** | **Reverse primer (5' → 3')** |
| --- | --- | --- |
| *CD86* | AAAGAGGAGCAAGCAGACGC | CTCCACGGAAACAGCATCTGAG |
| *CD206* | CTAACTGGGGTGCTGACGAG | GGCAGTTGAGGAGGTTCAGT |
| *IL-1β* | GCACGATGCACCTGTACGAT | CACCAAGCTTTTTTGCTGTGAGT |
| *TNF-α* | TGGAGCTGGCCGAGGAG | AGCAGGCAGAAGAGCGTGG |
| *iNOS* | GACATTACGACCCCTCCCAC | GCACATGCAAGGAAGGGAAC |
| *TGF-β* | TGATACGCCTGAGTGGCTGTCT | CACAAGAGCAGTGAGCGCTGAA |
| *IL-6* | TACCACTTCACAAGTCGGAGGC | CTGCAAGTGCATCATCGTTGTTC |
| *ARG1* | AATGAAGAGCTGGCTGGTGT | CTGGTTGTCAGGGGAGTGT |
| *OCN* | GGCGCTACCTGTATCAATGG | GTGGTCAGCCAACTCGTCA |
| *ALP* | CCGCCTCAGTGATTTAGGGC | GGGTCTGTAATCTGACTCTGTCC |
| *DC-STAMP* | GGCGAGTAGAGGTAGGGGTC | TAGGGGCCCCGCCAT |
| *TRAP* | GGCCAGCTTTGCCAAAATCAA | ATTGCTGAGGGGATGCAAGAG |
| *β-actin* | ATCCGTAAAGACCTCTATGC | AACGCAGCTCAGTAACAGTC |
